# Synthetic consortia towards photosynthetically derived acetate for heterotrophic production of 1-butanol

**DOI:** 10.1007/s00253-026-14029-z

**Published:** 2026-09-11

**Authors:** Stamatina Roussou, Peter Lindblad

**Affiliations:** Microbial Chemistry, Department of Chemistry-Ångström Laboratory, Uppsala University, Uppsala, Box 523, 75120 Sweden

**Keywords:** *Synechocystis* PCC 6803, *Escherichia coli*, *Pseudomonas taiwanensis*, Acetate, Consortia, 1-Butanol

## Abstract

**Abstract:**

Synthetic consortia is an emerging field in biotechnology, offering advantages such as division of labor between the individual members and reduced risk of contamination. Consortia combining phototrophic and heterotrophic bacteria are attractive, as phototrophs can utilize CO₂ and light energy to sustain growth. Several studies have explored such systems where sucrose is most commonly used as the provided carbon source. An alternative is acetate, a smaller two-carbon molecule produced as a by-product by many microorganisms, including the model cyanobacterium *Synechocystis* PCC 6803 (thereafter *Synechocystis*). Although acetate production during phototrophic growth is typically low, previous studies have demonstrated that metabolic engineering—specifically the introduction of phosphoketolase (PK) and overexpression of phosphotransacetylase (Pta)—enabled the development of a high-producing strain (WT_PKPa_RBS_BsPta_Δacs) capable of secreting significant levels of acetate into the medium (Roussou and Lindblad 2026). In this study, *Escherichia coli* and *Pseudomonas taiwanensis* were engineered to produce 1-butanol, an industrially relevant bulk chemical, and cultivated using acetate as the sole carbon source. These strains were then individually co-cultivated with the previously engineered *Synechocystis* strain, WT_PKPa_RBS_BsPta_Δacs, forming two distinct consortia that were maintained for 42 days. Growth dynamics were successfully monitored, and acetate concentrations in the consortia were lower than in a corresponding axenic *Synechocystis* culture. At the same time, 1-butanol production was detected in the two co-cultures, demonstrating the feasibility of coupling photosynthetically derived acetate to heterotrophic production of a value-added bulk chemical.

**Key points:**

**•** *Photosynthetic/heterotrophic synthetic consortia for 1-butanol production*.

**• ***Engineered Synechocystis PCC 6803 cells produce acetate from CO*_*2*_.

**• ***Modified Escherichia coli and Pseudomonas taiwanensis cells grow on acetate and produce 1-butanol*.

**Supplementary Information:**

The online version contains supplementary material available at https://doi.org/10.1007/s00253-026-14029-z.

## Introduction

It is common in nature to encounter microbial communities with established relationships between their members (Keller and Surette 2006). These relationships can, for example, be based on mutualism, commensalism, parasitism, or/and neutralism (Martin and Schwab 2013). Microbial communities have been studied not only for understanding reasons but also for biotechnological application, as it could be an alternative pathway to develop sustainable production (Jia et al. 2016). The symbiosis of microorganisms can be described by a variety of terms including co-cultures, mixed cultures, or synthetic consortia with the latter referring to the distribution of labor between different strains (Jia et al. 2016). This distribution of labor can reduce the metabolic burden imposed on individual strains. Furthermore, intermediates or substrates of the product biosynthetic pathway can be distributed among the strains (Shong et al. 2012). Another advantage of synthetic consortia is their potential for dynamic balance between the strains which can promote system stability through adaptation and long-term maintenance (Shong et al. 2012). Finally, a well-established multi-strains cultivation can be robust against contamination, offering a solution to a widespread problem when cultivating microorganisms with industrial purposes (Gao et al. 2022). Two main strategies can be applied to create artificial consortia: the top-down method and the bottom-up method (Großkopf and Soyer 2014). In the first case, a native consortium is re-engineered, through omics and in the second ration design is applied in order to create a new consortium (Wang et al. 2014; Slusarczyk et al. 2012).

A synthetic consortium can be created by different combinations of strains and species. Reported examples include single species consortia, containing different engineered strains of the same species (e.g., *Escherichia coli*—*E. coli*) or two different species (Eiteman et al. 2008). These systems can include combinations of different bacterial species, fungi and bacteria, or other microorganisms, for example archaeal-bacterial consortia (Shong et al. 2012; Marchand and Collins 2013). There are also reports of multiple species consortia, for example in wastewater treatment where co-cultivation of fungi, yeast, and microalgae was studied (Abdalla et al. 2024). Other examples of applications of synthetic consortia, in addition to wastewater treatment and production, are lignocellulose biorefineries and bioremediation (Qian et al. 2020).

An appealing type of consortium, based on symbiotic relationship, is between photoautotrophic and heterotrophic microorganisms. Photoautotrophs are microorganisms that have the ability to harvest light energy and use it to synthesize carbon metabolites from CO_2_ (Pathania and Srivastava 2025). Cyanobacteria are one of these photosynthetic microorganisms with the main carbon fixation pathway being the Calvin-Benson-Bassham (CBB) cycle (Liang et al. 2018). They are also capable of metabolic engineering and have been successfully engineered for the production of chemicals including fuels (Melis et al. 2024). This makes them excellent candidates for consortia, since they are able to synthesize and export carbon molecules synthesized by renewable source like the sunlight and the surplus of CO_2_ in the atmosphere while at the same time, they release O_2_. The carbon molecules can then be utilized by heterotrophs. The presence of heterotrophs can be beneficial for the cyanobacterium as it can provide more CO_2_ derived from the cell’s respiration as well as molecules like vitamins (Abinandan et al. 2018). Some cyanobacteria strains have been widely used in co-cultures, one of them is *Synechococcus elongatus* PCC 7942 (*S. elongatus* 7942) mostly because of its ability to overproduce sucrose while the heterotroph can be either *E. coli* or *Bacilus subtilis* (*B. subtilis*) or even *Saccharomyces cerevisiae* (*S. cerevisiae*) (Hays et al. 2017). Furthermore, *Synechococcus elongatus* UTEX 2973 (*S. elongatus* UTEX) have also been engineered to produce sucrose and was employed in a consortium with *E. coli* for the production of 3-hydroxypropionic acid (Zhang et al. 2020). Another microorganism that has been co-cultivated with cyanobacteria like *Synechococcus elongatus* or the model organism *Synechocystis* PCC 6803 (*Synechocystis*) is *Pseudomonas* sp., including *Pseudomonas taiwanensis* (*P. taiwanensis*) and *Pseudomonas putida* (*P. putida*) (Bozan et al. 2022; Kratzl et al. 2024). *Synechocystis* is one of the model organisms for the study of cyanobacteria and it has successfully been engineered for the production of various chemicals while it has also been employed in co-cultivation studies (Bolay et al. 2025; Tóth et al. 2022). Immobilized cells of *Synechocystis*, engineered to produce sucrose, were used to provide carbon to engineered *E. coli* for the transformation of cyclohexanone to ε-caprolactone (Tóth et al. 2022).

An alternative carbon source for co-culture can be the two-carbon molecule acetate (Kiefer et al. 2021), a natural bioproduct of the metabolism in many microorganisms, including *Synechocystis* (Phue et al. 2010; Du et al. 2018). Furthermore, a lot of microorganisms are able to grow on acetate as sole carbon source where it is catabolized to acetyl-CoA through the AckA-Pta or Acs pathway (Kiefer et al. 2021). *E. coli* can grow on acetate as a sole carbon source, with the strains *E. coli* W and *E. coli* BL21 DE (3) (*E. coli* BL21) showing better performance (Noh et al. 2018; Zhu et al. 2024). Another microorganism with capacity to grow on acetate is *Pseudomonas* sp. (Arnold et al. 2019). *P. putida* has been successfully cultivated on acetate as a source of carbon (Arnold et al. 2019) while another strain, *Pseudomonas taiwanensis* VLB120 (*P. taiwanensis* VLB120), can grow on a broad range of carbon sources including acetate (Neves et al. 2024). Both of these strains have shown positive impact on cyanobacteria when co-cultivated (Bozan et al. 2022; Kratzl et al. 2024).

**Fig. 1:**
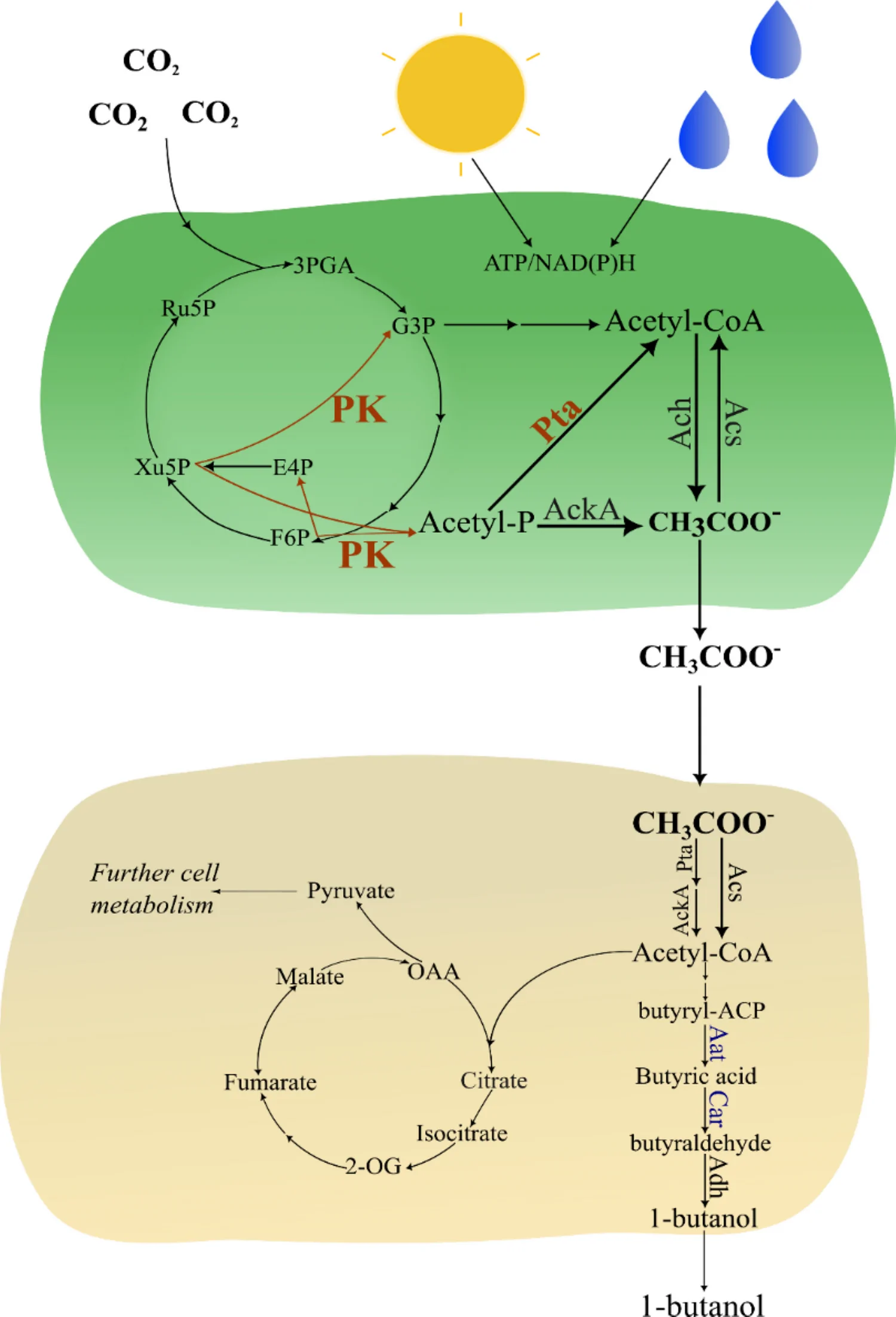
Simplified representation of the Calvin-Benson-Bassham (CBB) cycle and acetate biosynthesis pathways in the cyanobacterium *Synechocystis* PCC 6803 followed by a simplified representation of acetate assimilation by a heterotrophic bacterium (*Escherichia coli* or *Pseudomonas taiwanensis*) and 1-butanol production through the TPC pathway. Shown are also the starting molecule for the carbon assimilation for the cyanobacterium, CO_2_ as well as the solar energy and water, used to power the cell. The native pathways are represented with black solid lines while the solid orange lines represent the inserted phosphoketolase. The intermediates of CBB and TCA cycle are shown through abbreviations; 3PGA, 3-phosphoglycerate; G3P, glyceraldehyde-3-phosphate; F6P, fructose-6-phosphate; E4P, erythrose-4-phosphate; Xu5P, xylulose-5-phosphate; Ru5P, ribulose-5-phosphate; OAA, oxaloacetate; 2-OG, 2-oxoglutarate. The following enzymes are also shown; Pta, phosphotransacetylase; AckA, acetate kinase; Acs, acetyl-CoA synthetase; Ach, acetyl-CoA hydrolase; PK, phosphoketolase; Aat, acyl-ACP thioesterase; Car, carboxylic acid reductase; Adh, aldehyde reductase. PK and Pta are shown in orange since they were expressed heterogeneously to promote acetate biosynthesis. The enzymes Aat and Car are shown in blue since they expressed heterogeneously for 1-butanol production. The acetate is shown by its chemical form CH_3_COO^−^

In the present study, we used a previously engineered *Synechocystis* strain capable of producing high levels of acetate through the insertion of phosphoketolase and overexpression of phosphotransacetylase (Roussou et al. 2025; Roussou and Lindblad 2026). The expression of these two enzymes creates a bypass between the main carbon fixation pathway, the CBB-cycle, and acetate formation. The resulting strain, WT_PKPa_RBS_BsPta_Δacs, is proven to reach high yields of acetate (up to 2.7 g/L in flask cultivation). This strain was used as the carbon source provider (farmer) in two different synthetic consortia, one based on *E. coli* BL21 and a second based on *P. taiwanensis* VLB120 (Fig. 1). Both of the heterotrophs were engineered to produce 1-butanol, a bulk chemical with numerous industrial applications as well as being an excellent fuel (Jin et al. 2011). The aim of the study was to investigate if acetate generated from CO_2_ by photosynthetic *Synechocystis* cells as a carbon source can support the growth of the heterotrophs as well as the synthesis of 1-butanol. We observed growth of both heterotrophs in the respective consortium and, even though the 1-butanol productions were low, the results demonstrate the potential of acetate-based consortia for photosynthetically driven production of valuable chemicals from CO_2_.

## Materials and methods

### Strains and cultivation methods

An engineered strain of the unicellular cyanobacterium *Synechocystis* PCC 6803 (*Synechocystis*, strain WT_PKPa_RBS_BsPta_Δacs), able to achieve high yield of acetate, was used as farmer in this study (Roussou and Lindblad 2026). The temperature was 30 °C and the light intensity was set at 65 μmol photons m^−2^s^−1^. The pre-culture of the strain was in BG11 medium (Stanier et al. 1971) while for the co-culture, an optimized version of BG11 was used (CoBG11); BG11 supplied with 4 mM NH_4_Cl, 13.1 mM (3 g/L) TES (pH adjusted 8.0) and 150 mM NaCl (Sigma-Aldrich) (Zhang et al. 2020).

The heterotrophic strains used in this study were *Escherichia coli* BL21 DE3 (*E. coli* BL21) and *Pseudomonas taiwanensis* VLB120 (*P. taiwanensis* VLB120) while *E. coli* T7 was used as the subcloning strain. All heterotrophic strains were cultivated in LB medium. In addition, *E. coli* BL21 and *P. taiwanensis* VLB120 were cultivated in CoBG11 medium supplied with sodium acetate (SoAc) (Sigma-Aldrich), except when co-cultivated with the cyanobacterium where no additional carbon source was used. Both strains were cultivated at 30 °C with the exception of the transformation experiments for the *E. coli* strains, where the strains were cultivated at 37 °C.

All of the strains were cultivated either in liquid cultures or on agar plates supplied with 1.5% w/v agar. The antibiotic used was kanamycin (Sigma-Aldrich) in a final concentration of 50 μg/mL.

### Growth analysis

The growth in the monocultures was monitored through optical density (OD) measured by a Varian Cary 50 BIO spectrophotometer. For *Synechocystis*, the OD was measured in 750 nm while for the other two strains, in 600 nm. In the synthetic consortia experiment, the growth of *P. taiwanensis* VLB120 and *E. coli* BL21 was determined by viable counts plated on LB agar plates with the corresponding antibiotic and it was monitored as CFU per mL. The cells were serially diluted in sterile H_2_O and 10 μl of each dilution was plated on the LB agar plate. Then the cells were incubated overnight in the optimal temperature for each strain (37 °C for *E. coli* BL21 and 30 °C for *P. taiwanensis* VLB120, respectively).

To follow the growth of *Synechocystis* in the consortium, chlorophyll extraction was performed. Chlorophyll a and carotenoids were extracted from 2.5 mL sampled from the consortium. The cultures were centrifuged for 10 min at maximum speed. The supernatant was used for acetate and 1-butanol analyses and the pellet was resuspended in 5 mL cold methanol (+4 °C) before it was homogenized by vortex and incubated for 1 h at + 4 °C. After a second centrifugation, the absorbances were measured at A470, A665, and A720. The final chlorophyll a concentration and carotenoids were calculated by the equations by Ritchie (2006) and Wellburn (1994), respectively. The protocol was adjusted from Roussou et al. 2025.

### Construction of plasmids and transformation of the heterotrophs

In order to engineer the two heterotrophs to produce 1-butanol, a synthetic O_2_-tolerant butanol pathway was used, TPC4 (Pásztor et al. 2015). The genes constituting the pathway were amplified from the plasmid pET-TPC4 with specific primers and cloned into the plasmid pPMQAK1 (Huang et al. 2010). The promoters tested were the thiolase promoter from *Clostridium acetobutylicum* (Collas et al. 2012) and the P*em7* synthetic promoter which was ordered as a gBLock (Table S.1 b) (Zobel et al. 2015). The gBLock contains an optimized bicistronic design (BCD2) (Mutalik et al. 2013) aiming to optimize the translation of the pathway. Finally in both cases, a FLAG taq was added in the construct as well, expressing in the C-terminus of the first protein. The promoters and the expression cassettes (Figure S.1) were joined through overlap extension PCR, while the final product was digested with *Xba*I and *Pst*I restriction enzymes. The plasmid was also digested with the same enzymes. The resulted fragments were ligated and transformed into competent *E. coli* T7 through heat-shock transformation. Colony PCR and sequencing for verification were followed. The correct plasmid was then transformed into *E. coli* BL21 as above, while for *P. taiwanensis* VLB120, electroporation was used as the transformation method.

The primers and gBLock used in this study are shown in Table S.1 and the corresponding strains produced in Table S.2. When DNA fragments were amplified or joined through overlap extension PCR, the polymerase used was Phusion DNA polymerase (Thermo Fisher Scientific) while for colony PCR, DreamTaq DNA polymerase was used (Thermo Fisher Scientific).

### Synthetic consortia experiments

Pre-cultures of all the strains were started from glycerol stocks. The *Synechocystis* strain was cultivated in 6-well TC plates (Sarstedt) until mid-log phase using BG11 supplemented with 50 μg/mL kanamycin. Then, the culture was used to start pre-cultures in 250-mL Erlenmeyer flasks (VWR) in a total volume of 50 mL CoBG11 containing the antibiotic and 50 mM NaHCO_3_. The experimental triplicates were started at OD_750_ = 0.03 in a total volume of 25 mL in Biolite 25 cm^2^ plug-sealed tissue culture flasks (Thermo Fisher Scientific) in the same medium composition as in the pre-culture.

For the heterotrophic strains, adaptive laboratory evolution (ALE) was performed to improve growth on acetate as the sole carbon source. All subsequent experiments were performed in triplicate. Starting from pre-cultures in LB medium, cells were inoculated into CoBG11 supplemented with 2 g/L SoAc at an initial OD_600_ of 0.05 and cultivated for 6 days. In order to inoculate the cells in CoBG11 medium, the cells were concentrated and resuspended in CoBG11 to remove any remaining LB medium. After this adaptation period, fresh cultures were established at the same initial OD_600_ (0.05) while the SoAc concentration in the CoBG11 medium was reduced to 1 g/L. At the 6^th^ day, 10% of the culture was used to inoculate a fresh culture. On day 6, 10% (v/v) of each culture was used to inoculate fresh medium. This transfer process was repeated five times, with each subsequent cultivation lasting 2 days, and glycerol stocks were prepared after the fifth re-inoculation. These glycerol stocks were subsequently used to establish two fresh series of cultures (initial OD_600_ = 0.05), where growth was evaluated in CoBG11 supplemented with either 1 g/L or 0.5 g/L SoAc. Glycerol stocks from the cultures that grew in CoBG11 supplemented with 0.5 g/L SoAc were prepared. These glycerol stocks, with the adapted cells growing with 1 g/L SoAc, were used to inoculate pre-cultures for the synthetic consortia experiment. The pre-cultures took place in 250-mL Erlenmeyer flasks (VWR) in a total volume of 50 mL CoBG11 containing the antibiotic and supplemented with 0.5 g/L SoAc.

The three triplicate *Synechocystis* cultures were started on day −2 and on day 0 of the consortium experiment; in two triplicates of *Synechocystis* (OD_750_ = 2.5), the heterotrophic cells were added (starting OD_600_ = 0.5). The final experiment contained a *Synechocystis* monoculture in triplicate, a triplicate containing *Synechocystis* and *E. coli* BL21, and a triplicate of *Synechocystis* and *P. taiwanensis* VLB120.

Every second day, 2.5 mL of culture was removed and 2.5 mL of fresh CoBG11 medium supplemented with kanamycin and 500 mM NaHCO_3_ was added. On the same day, the pH was adjusted to 7 through the use of 37% HCl (Sigma-Aldrich). After day 30, this routine was repeated every 4 days. The samples from the cultures were used for OD_750_ measurements (for the monoculture), viable counts (for the heterotrophs), chlorophyll extraction on selected days, and 1-butanol and acetate quantifications.

### Acetate and 1-butanol quantification

In order to quantify the production of 1-butanol, a previously established method was used (Liu et al. 2019). In summary, 2.07 mL supernatant was transferred in a 15-mL screw cap tube and the internal standard (30 µl of 7000 mg/L isobutanol) and extraction solvent (700 µl DCM, dichloromethane) were added. Then, the tube was shaken in a multi-tube Vortexer VX-2500 (VWR) for 5 min and centrifugation followed for clearer separation of the two phases. The DCM phase was transferred into a gas chromatography (GC) vial and 1-butanol was quantified through gas chromatography. The acetate remained in the H_2_O phase so the next step was to leave the tube overnight in the fume hood, so any leftover DCM could evaporate and then the acetate measurements took over as described earlier (Roussou et al. 2025; Roussou and Lindblad 2026). The H_2_O phase was filtered with a 0.22-µm filter and 0.5 mL was transferred to an NMR tube. Also added was the internal standard, 10 μl of 50 μM HOOCC_6_H_4_COOK and D_2_0 to a final volume of 0.6 mL. All chemicals used were from Sigma-Aldrich.

## Results

### Escherichia coli can grow on acetate as carbon source and produce 1-butanol

Acetate can serve as a carbon source for microorganisms such as *E. coli*. Among the different *E. coli* strains, *E. coli* BL21 has shown considerable potential for growth on acetate as a sole carbon source (Zhu et al. 2024). This strain was engineered to produce 1-butanol through diversion of the fatty acid synthesis pathway (Pásztor et al. 2015), an oxygen-tolerant pathway. In the first step, the pathway was expressed using a self-replication vector (pPMQAK1), under the thiolase promoter (P*thl*) with kanamycin as selected antibiotic marker.

**Fig. 2:**
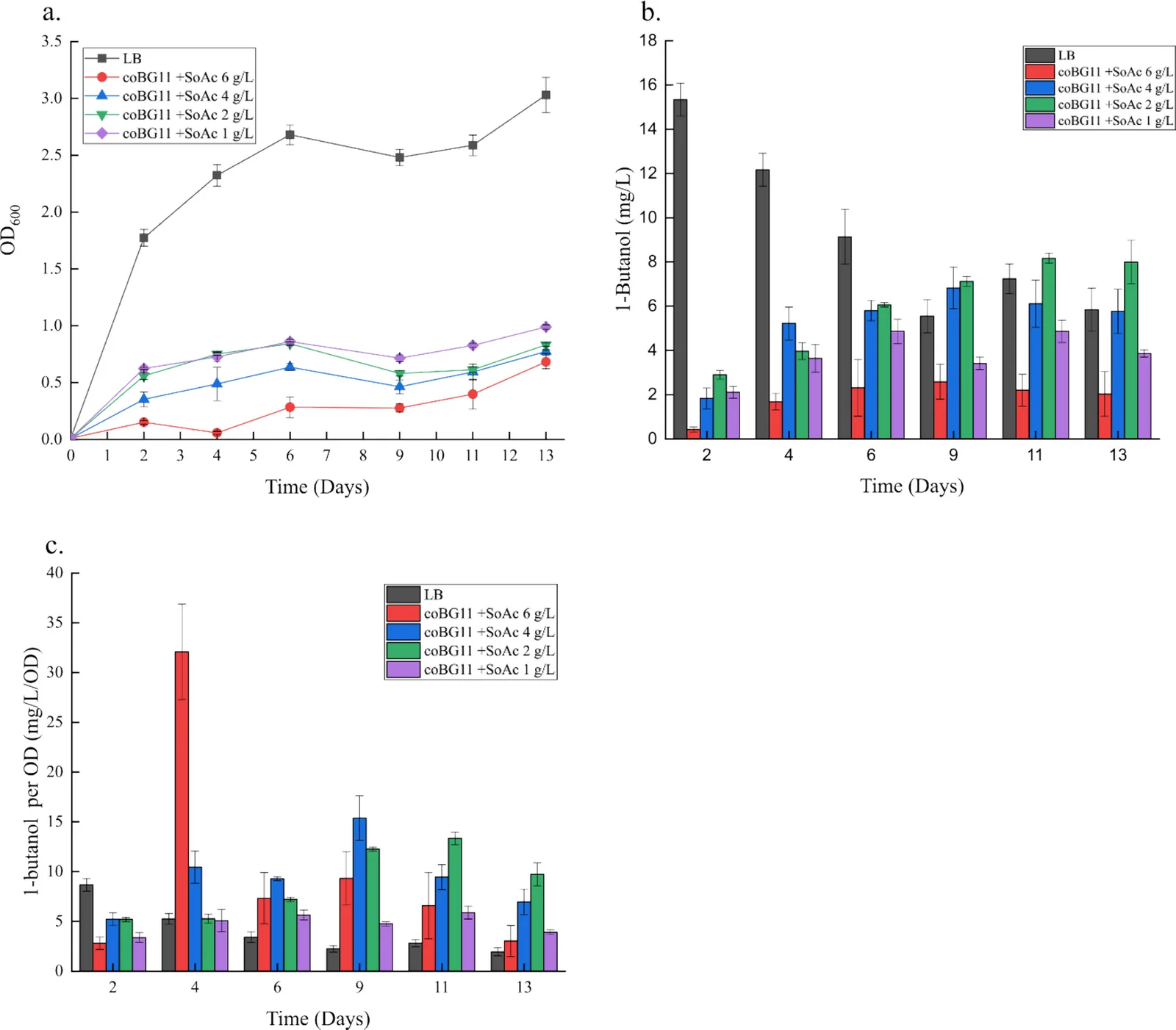
Growth (presented by OD_600_) of *E. coli*_Pthl_ButOH (**a**) and 1-butanol production under LB medium and CoBG11 supplied with different concentrations of sodium acetate as the carbon source. The butanol production is shown both per volume (**b**) and normalized by the OD (**c**). On every sampling point (days 2, 4, 6, 9, 11, 13), 10% of the culture was sampled and replaced with the respective medium. The results are represented as mean ± SD of three biological replicates

To investigate the ability of the strain to grow on acetate as a carbon source, it was cultivated in a minimum medium supplemented with SoAc in various concentrations. The medium chosen was an optimized version of BG11 (CoBG11) in order to also investigate if it would be feasible to grow *E. coli* in a medium suitable for cyanobacteria (Hays et al. 2017). Four different concentrations of SoAc were chosen: 6, 4, 2, and 1 g per liter. The bacteria were grown in 25 mL volume in a closed system and samples (10% of the volume) were taken at the days 2, 4, 6, 9, 11, and 13, except for the last day of the experiment, and the sampling volume was replaced with fresh medium containing acetate at the corresponding concentration. Growth and 1-butanol production of the strain in the different conditions are shown in Fig. 2. The experiment demonstrated that the lower the concentration of the added acetate, the better the growth of the strain. It is also clear that the growth of the cells in CoBG11 with acetate is generally much lower compared to when grown in LB medium, a rich medium for heterotrophs (Fig. 2a). After day 9, further growth was observed in cultures using acetate as the sole carbon source, whereas no further growth was observed in the culture grown in LB medium. It was possible to demonstrate 1-butanol production under all cultivation conditions examined. During the first days of the experiment, the highest production was observed in the strain grown in LB medium. However, during the final three sampling points, higher 1-butanol production was observed in strains grown with the addition of 4 and 2 g/L acetate, with the 2 g/L acetate condition showing the highest production levels on days 11 and 13 (Fig. 2b). When 1-butanol production was normalized per OD, the titers are higher in the strains growing with acetate as the carbon source (Fig. 2c). A similar trend is shown for the strain growing with the addition of 2 g/L acetate with the highest 1-butanol production per OD during the last two days of the experiment.

### Enhanced growth and 1-butanol production of E. coli and P. taiwanensis in minimum acetate concentrations through repeated cultivation

**Fig. 3:**
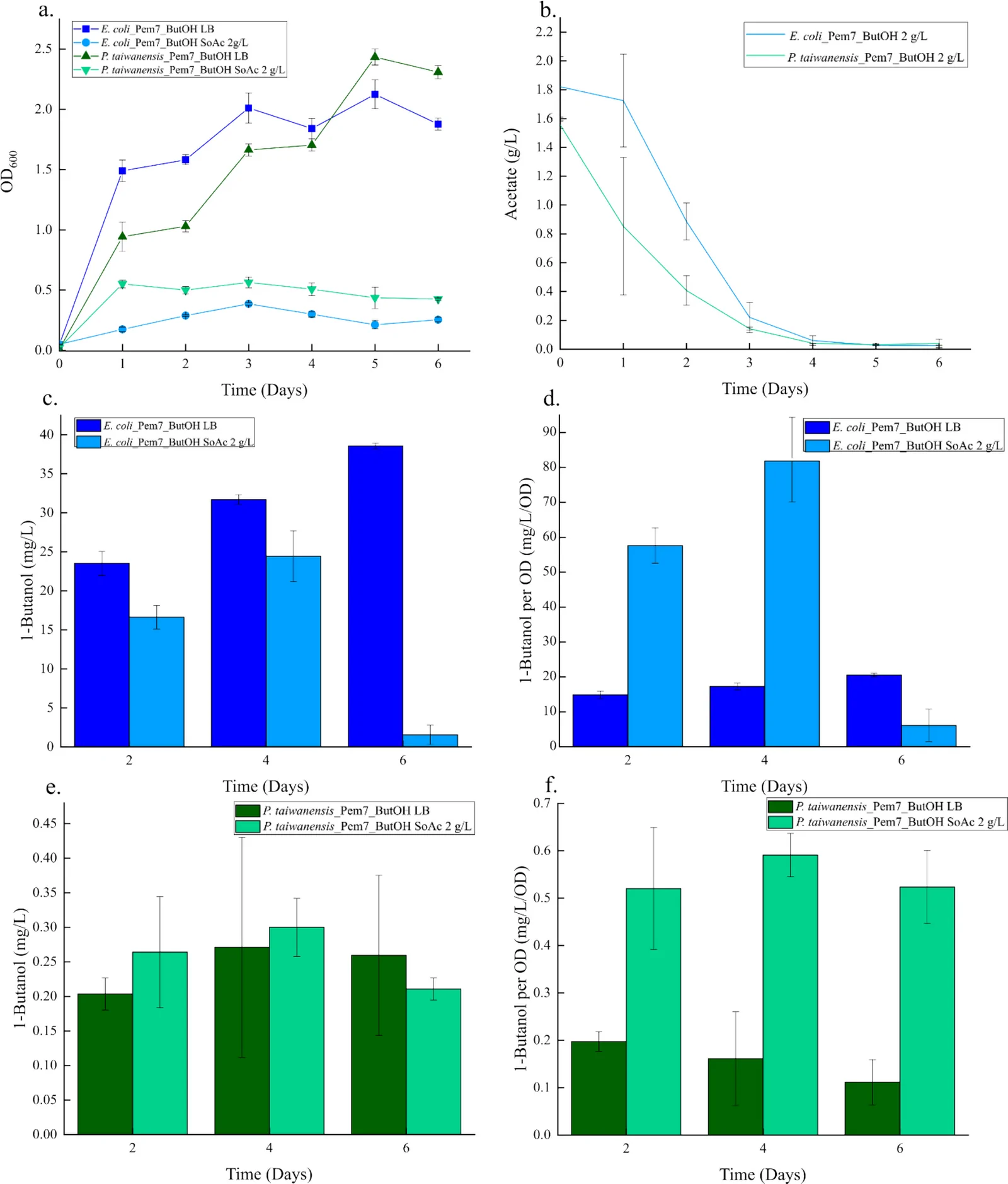
Growth and 1-butanol production of *E. coli*_Pem7_ButOH and *P. taiwanensis*_Pem7_ButOH under LB medium (LB) and CoBG11 medium supplied with 2 g/L sodium acetate (SoAc 2 g/L). The growth is shown by the OD_600_ (**a**) and the acetate consumption through the experiment is shown (**b**). The 1-butanol volumetric titers (**c** and **e**) and normalized by the OD (**d** and **f**) are shown for both bacteria. The cultures were sampled every 2 days for 6 days and the volume was not replaced. The results are represented as mean ± SD of three biological replicates

Another bacterium that can grow efficiently on acetate as a sole carbon source is *P. taiwanensis* VLB120 (Neves et al. 2024). The strain was engineered with the same self-replication vector resulting in the strain *P. taiwanensis*_Pthl_ButOH. In order to optimize 1-butanol production, the promoter was switched to a synthetic promoter with strong expression in both *E. coli* BL21 and *P. taiwanensis* VBL120, the P*em7* promoter (Zobel et al. 2015). The new strains, *E. coli*_Pem7_ButOH and *P. taiwanensis*_Pem7_ButOH, showed slightly higher production in screening experiments (Figure S.2). The strains were initially cultivated in 25 mL volume in closed systems for 6 days with sampling (2.5 mL) every second day. The starting OD was set as OD_600_ = 0.05 for both microorganisms. The sampled volumes were not replaced, as the aim was to monitor the growth and 1-butanol production of the cells under a specific starting condition. The control triplicate was grown in LB medium while the experimental in CoBG11 supplied with 2 g/L SoAc. The growth is shown in Fig. 3a and it can be observed that *P. taiwanensis*_Pem7_ButOH grew better than *E. coli*_Pem7_ButOH when cultivated in acetate as carbon source. In Fig. 3b the acetate consumption throughout the experiment is shown where *P. taiwanensis*_Pem7_ButOH seems to consume the acetate at a more stable rate. 1-Butanol was generated by both bacteria (Fig. 3c–f). In the case of *E. coli*_Pem7_ButOH, the volumetric 1-butanol production was higher for the culture growing in LB medium compared to the culture growing in CoBG11 supplemented with 2 g/L SoAc. When the production is normalized per OD, the strain produced more 1-butanol per OD in CoBG11 supplemented with 2 g/L SoAc for the first two sampling points while on the last day, the production per OD was very low both per volume and per OD. In the case of *P. taiwanensis*_Pem7_ButOH, the 1-butanol production was similar in LB and in CoBG11 containing 2 g/L SoAc. Important to mention is that in the first two sampling points, the production was higher when acetate was the carbon source. Finally, the production per OD was always higher when the cultures were growing with acetate compared to the cultures growing in LB medium.

**Fig. 4:**
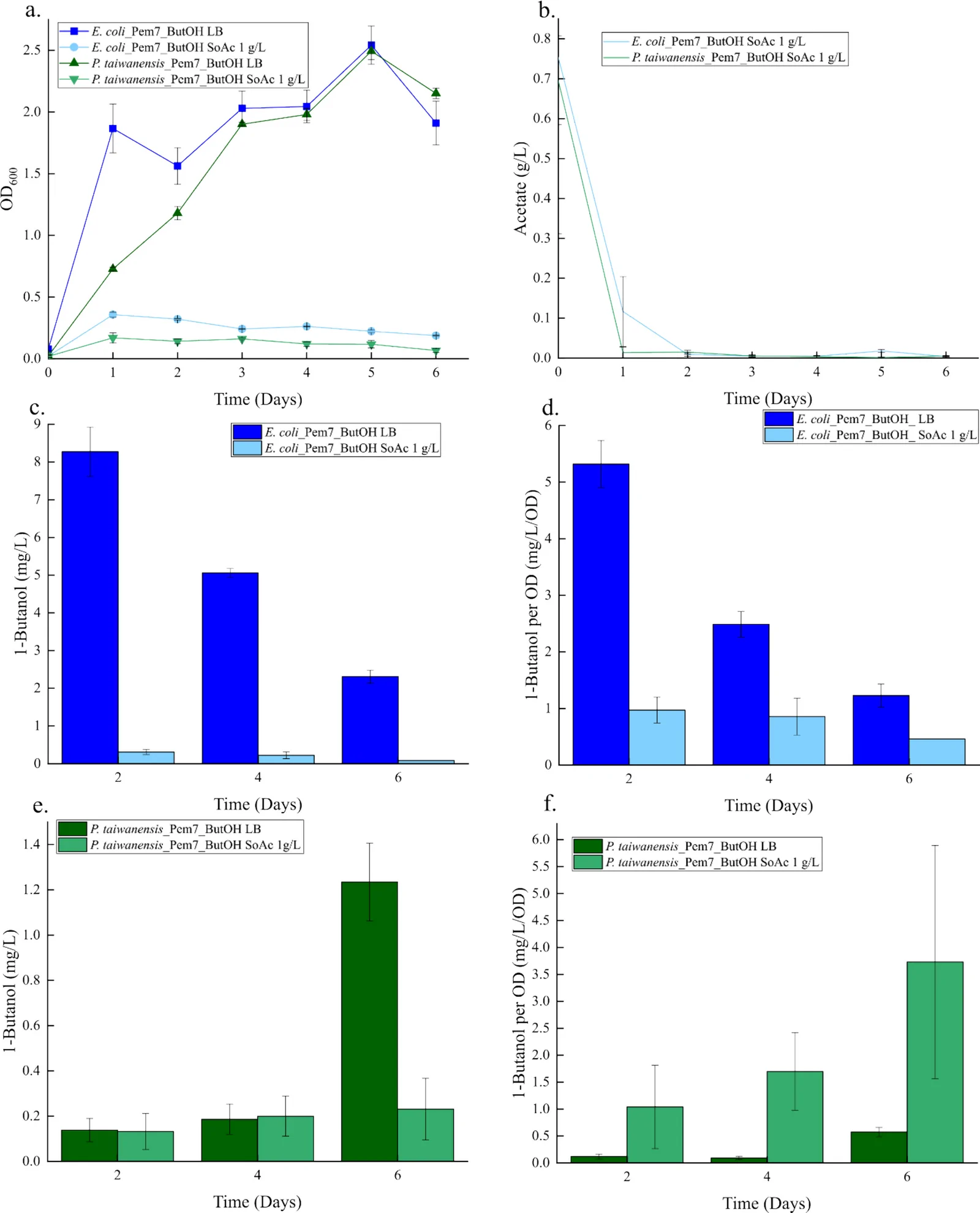
Growth and 1-butanol production of *E. coli*_Pem7_ButOH and *P. taiwanensis*_Pem7_ButOH under LB medium (LB) and CoBG11 medium supplied with 1 g/L sodium acetate (SoAc 1 g/L). The growth is shown by the OD_600_ (**a**) and the acetate consumption throughout the experiment is shown (**b**). The 1-butanol volumetric titers (**c** and **e**) and normalized by the OD (**d** and **f**) are shown for both bacteria. The cultures were sampled every 2 days for 6 days and the volume was not replaced. The results are represented as mean ± SD of three biological replicates

In the last day of the experiment (the 6^th^ day), the cultures were used to inoculate (starting OD_600_ = 0.05) a new round of experiment, where the acetate addition was reduced to 1 g/L. In this experiment, it is noticeable that the *E. coli*_Pem7_ButOH grew better in both media (Fig. 4a). The acetate consumption was also more stable in the case of *E. coli*_Pem7_ButOH (Fig. 4b). For the 1-butanol production, the same pattern as the previous experiment was observed but the level of titers was higher when the medium was LB for the *E. coli*_Pem7_ButOH (Fig. 4c) even though the production per OD was higher in the LB medium compared to when using CoBG11 with the addition of 1 g/L SoAc (Fig. 4d). In the case of *P. taiwanensis*_Pem7_ButOH, the 1-butanol production was stable in both media and followed the same pattern for both volumetric and per OD as in the previous growth condition (Fig. 4e, f).

**Fig. 5:**
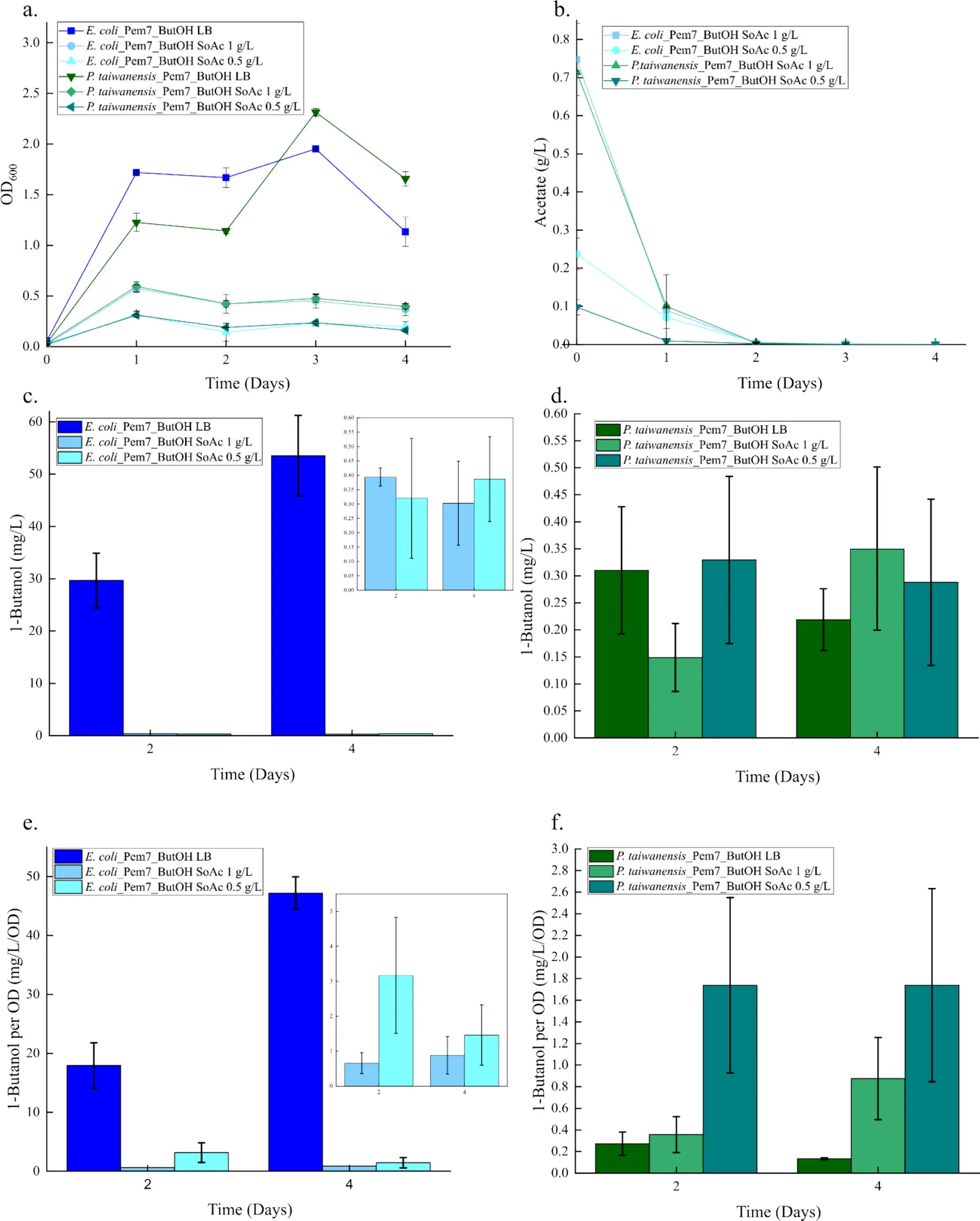
Growth and 1-butanol production of *E. coli*_Pem7_ButOH and *P. taiwanensis*_Pem7_ButOH under LB medium (LB) and CoBG11 medium supplied with 1 g/L sodium acetate (SoAc 1 g/L) or 0.5 g/L sodium acetate (SoAc 0.5 g/L). The growth is shown by the OD_600_ (**a**) and the acetate consumption throughout the experiment is shown (**b**). The 1-butanol volumetric titers (**c** and **e**) and normalized by the OD (**d** and **f**) are shown for both bacteria. The cultures were sampled every 2 days for 4 days and the volume was not replaced. The results are represented as mean ± SD of three biological replicates

Since the 1-butanol production in LB medium decreased significantly between the two re-inoculations, the experimental design was revised, and for the next round of experiments, the re-cultivation in LB medium was not included. The cultures growing on 1 g/L SoAc were used to inoculate (10% v/v) new cultures containing CoBG11 SoAc 1 g/L in order to promote the growth adaptation of the strain in acetate as a sole carbon source. After 2 days of cultivation, new sub-cultures were started using again 10% inoculum. This was repeated in total five times. At the last day of the experiment, glycerol stocks were made for both bacteria. A new experiment was then set up using glycerol stocks of the new strains for both media. This time, the strains were cultivated in LB, CoBG11 with the addition of SoAc 1 g/L, and CoBG11 with the addition of SoAc 0.5 g/L and a starting OD_600_ of 0.05 (Fig. 5). The cultivation lasted for 4 days, until the end of increase in the growth of the strains (Fig. 5a). The cultures already adjusted to grow in 1 g/L SoAc reached higher OD values compared to the previous experiment, reaching almost OD_600_ = 0.5 for both bacteria while we also noticed growth in the cultures supplied with 0.5 g/L SoAc. The acetate was consumed very fast in all cases to be non-detectable after day 2 (Fig. 5b). The 1-butanol production for *E. coli*_Pem7_ButOH during growth in LB medium was back to the levels of the first experiment while the 1-butanol production for the growth in 1 g/L SoAc did not improve and 1-butanol production was detected when the culture was supplied with 0.5 g/L SoAc (Fig. 5c). The 1-butanol production for the culture supplied with 0.5 g/L showed overall similar levels of 1-butanol production both in volumetric and normalized per OD compared to the culture supplied with 1 g/L SoAc (Fig. 5c, d). The 1-butanol titer for *P. taiwanensis*_Pem7_ButOH did not increase compared to the non-adjusted culture but again, the production between the two cultures growing with acetate was overall similar (Fig. 5e) and when normalized per OD, the production is higher in the case of the culture supplied with 0.5 g/L (Fig. 5f).

### Towards photosynthetically produced 1-butanol from synthetic consortia between E. coli or P. taiwanensis and an acetate-producing cyanobacterium

It was possible to plan a synthetic consortium experiment based on acetate as the provided carbon source since photosynthetic *Synechocystis* strains with enhanced acetate production were developed (Roussou and Lindblad 2026). The heterotrophic strains selected originated from the last growth experiment that showed increased OD_600_ values when cultivated with 1 g/L acetate as a sole carbon source. Precultures of *E. coli*_Pem7_ButOH and *P. taiwanensis*_Pem7_ButOH were set up and the medium was CoBG11 supplied with 1 g/L SoAc. At the same time, a pre-culture of the previously engineered cyanobacterium strain overproducing acetate also started. This strain was expressing PKPa and BsPta (WT_PKPa_RBS_BsPta_Δacs) and showed stable high acetate production throughout long-term cultivation (Roussou and Lindblad 2026). The strain was selected as the farmer strain for a synthetic consortium based on acetate as the carbon source. The pre-culture was grown with CoBG11 supplemented with 50 mM NaHCO_3_ and kanamycin. The experimental culture volume was 25 mL and closed system was applied since 1-butanol is volatile. Three biological triplicates were set up with starting OD_750_ of 0.03 and after 2 days of cultivation, the synthetic consortium was started. In one triplicate, *E. coli*_Pem7_ButOH with OD_600_ = 0.5 was added and in second *P. taiwanensis*_Pem7_ButOH with the same starting OD_600_, and the last third triplicate was a monoculture of the cyanobacterium to monitor the acetate production in the same growth conditions. The OD_750_ of the cyanobacterium was 2.5, so the ratios of ODs between the organisms were 5 to 1.

**Fig. 6:**
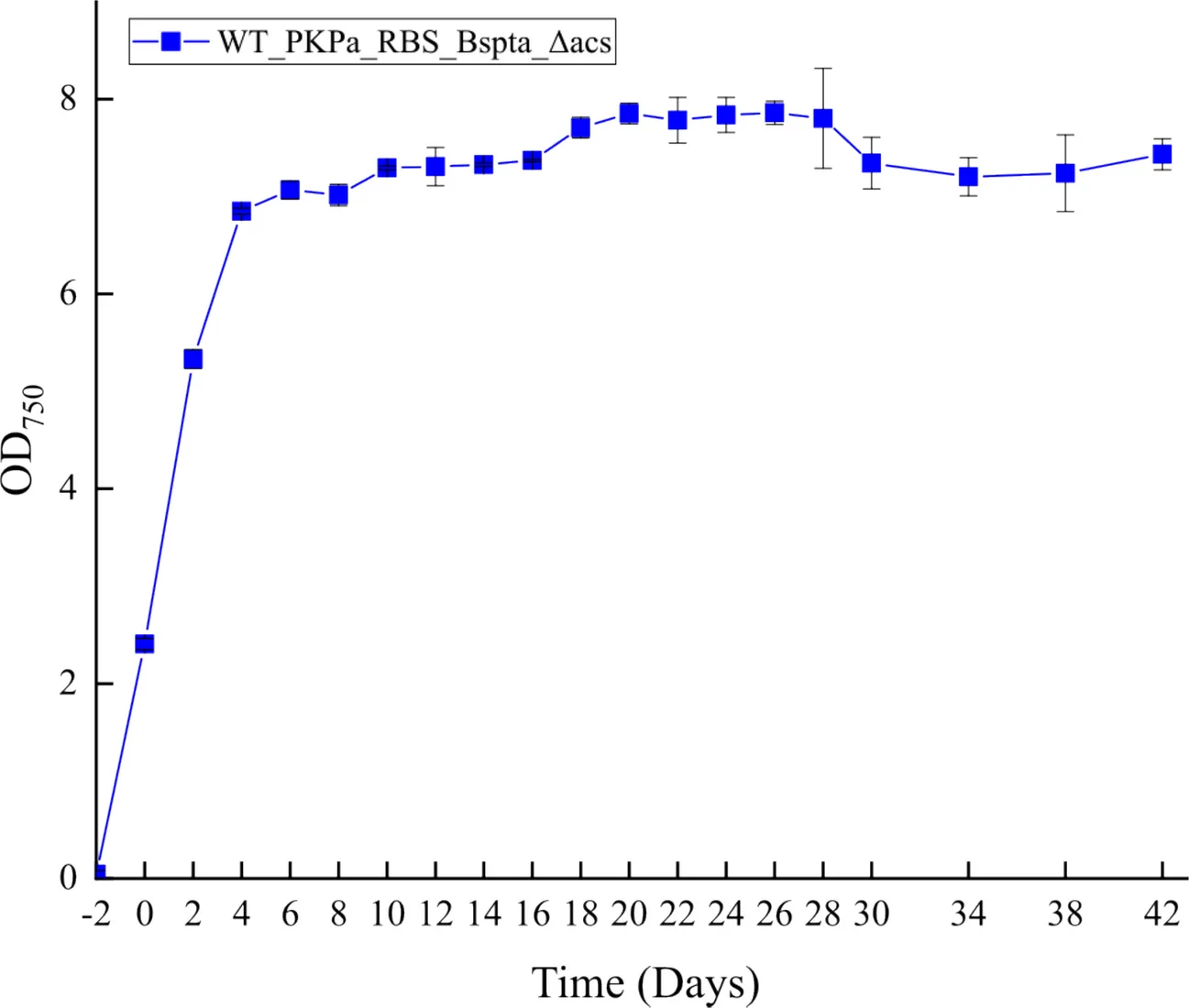
Growth of the cyanobacterium *Synechocystis* PCC 6803 engineered with PKPa and BsPta organized as a single operon in locus *acs* (WT_PKPa_RBS_BsPta_Δacs). The growth is shown as OD_750_. The light intensity was 65 μmol photons m^−2^s^−1^ and the temperature was 30 °C. 10% of the volume (2.5 mL) was sampled every 2 days from day −2 to day 30 while from day 30 to day 42, the culture was sampled every 4 days and replaced with 2.5 mL of CoBG11 containing 500 mM NaHCO_3_ (final concentration 50 mM) and kanamycin and the pH was adjusted at 7 with the addition of 37% HCl. On day 0, the heterotrophs were added at the respective triplicate of cyanobacterium and the co-cultivation started; as day −2 we define the day we started the growth of cyanobacterium. The 2.5 mL was used for OD, acetate measurements and on specific dates for chlorophyll measurements. The results are represented as mean ± SD of the three biological replicates

**Fig. 7:**
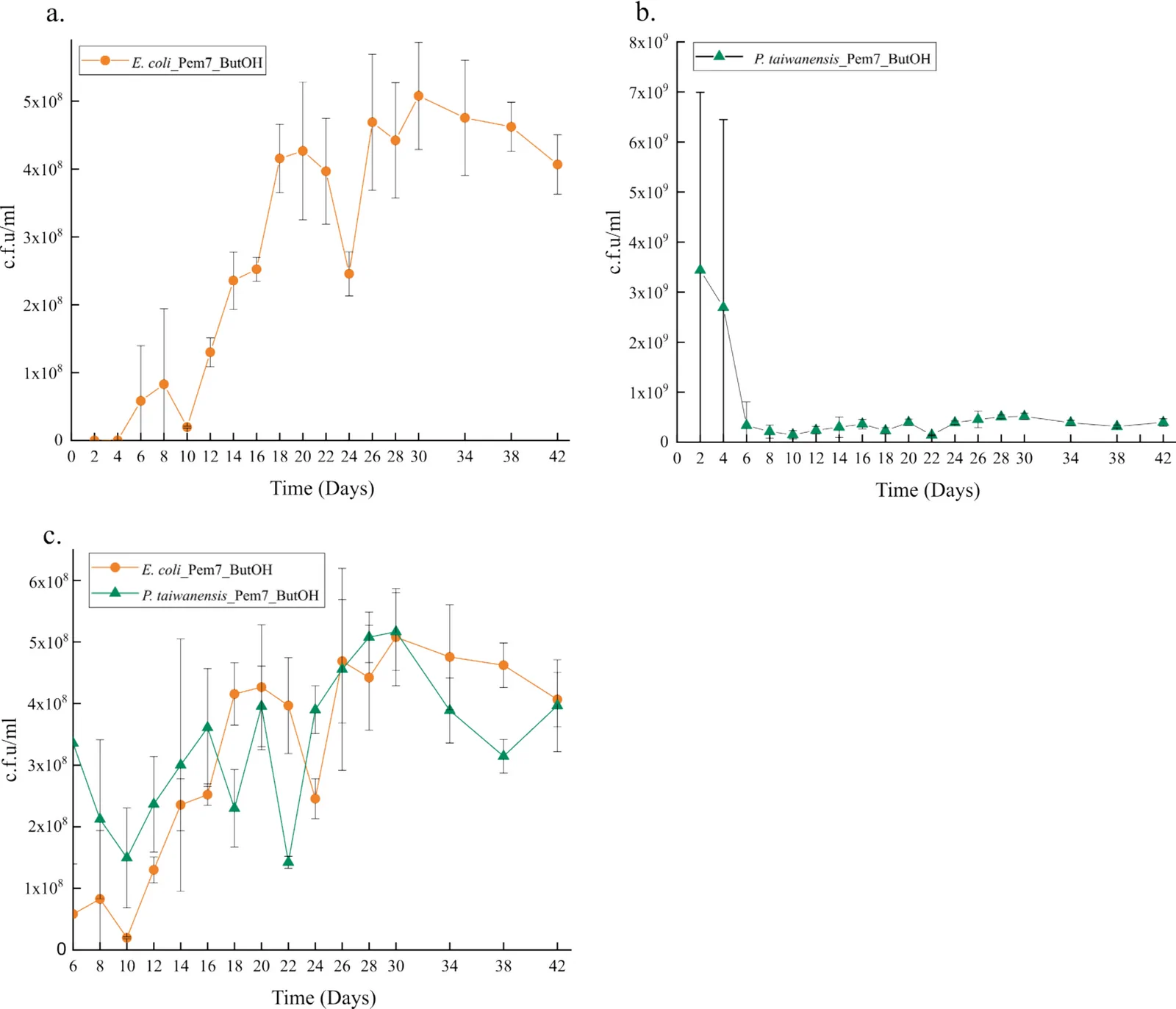
Growth of *E. coli*_Pem7_ButOH (**a**) and *P. taiwanensis*_Pem7_ButOH (**b**) during the synthetic consortia with WT_PKPa_RBS_BsPta_Δacs and a comparison between the two (**c**). The consortia were growing under light intensity 65 µmol photons m^−2^s^−1^ and the temperature was 30 °C. The growth is shown as c.f.u per mL, measured every 2nd day until day 30 and every 4th day until day 42. 2.5 mL was sampled on these days and 20 µl of the sample was used for serial dilutions and platted on LB Km^50^ agar plates. The sampling volume was replaced with equal volume (2.5 ml) of CoBG11 containing 500 mM NaHCO_3_ (final concentration 50 mM) and kanamycin and the pH was adjusted at 7 with the addition of 37% HCl. The rest was used for 1-butanol and acetate quantification and on selected days for chlorophyll measurements. The results are represented as mean ± SD of the three biological replicates

For simplicity, day 0 is defined as the day the consortium started. Between day 0 and day 30, the cultures were sampled every second day; 10% of the volume was removed and replaced with fresh CoBG11 containing 500 mM NaHCO_3_ (final concentration 50 mM) and kanamycin, and the pH adjusted to 7 with the addition of 37% HCl. After day 30, the sampling and replacement took place every 4th day. The growth of the monoculture of WT_PKP a_RBS_BsPta_Δacs is shown in Fig. 6; the culture entered stationary phase after day 6. To monitor the growth of the heterotrophs, viable counts were applied (Fig. 7). When *E. coli*_Pem7_ButOH was cultivated with WT_PKPa_RBS_BsPta_Δacs, very few cells were observed on days 2 and 4. On days 6 and 8, the system still showed instability. However, after day 10, the cell numbers started to increase (with a drop on day 24) and slowly decreased after day 30. In the consortium based on *P. taiwanensis*_Pem7_ButOH and WT_PKPa_RBS_BsPta_Δacs, there were a lot of cells in the first 4 days but again the system showed instability and stabilized only after day 6. If both of the heterotrophs are plotted together after day 6, where stability has been reached, several observations can be noted. There is a significant drop of *P. taiwanensis*_Pem7_ButOH cells on day 22 which was recovered between day 24 and day 30. Additionally, a second drop was followed, with indication of recovery on day 42. On the first days of the experiment, the same bacterium showed higher growth compared to *E. coli*_Pem7_ButOH (days 6–16) while after that, the population of *P. taiwanensis*_Pem7_ButOH remained lower compared to the other bacterium.

**Fig. 8:**
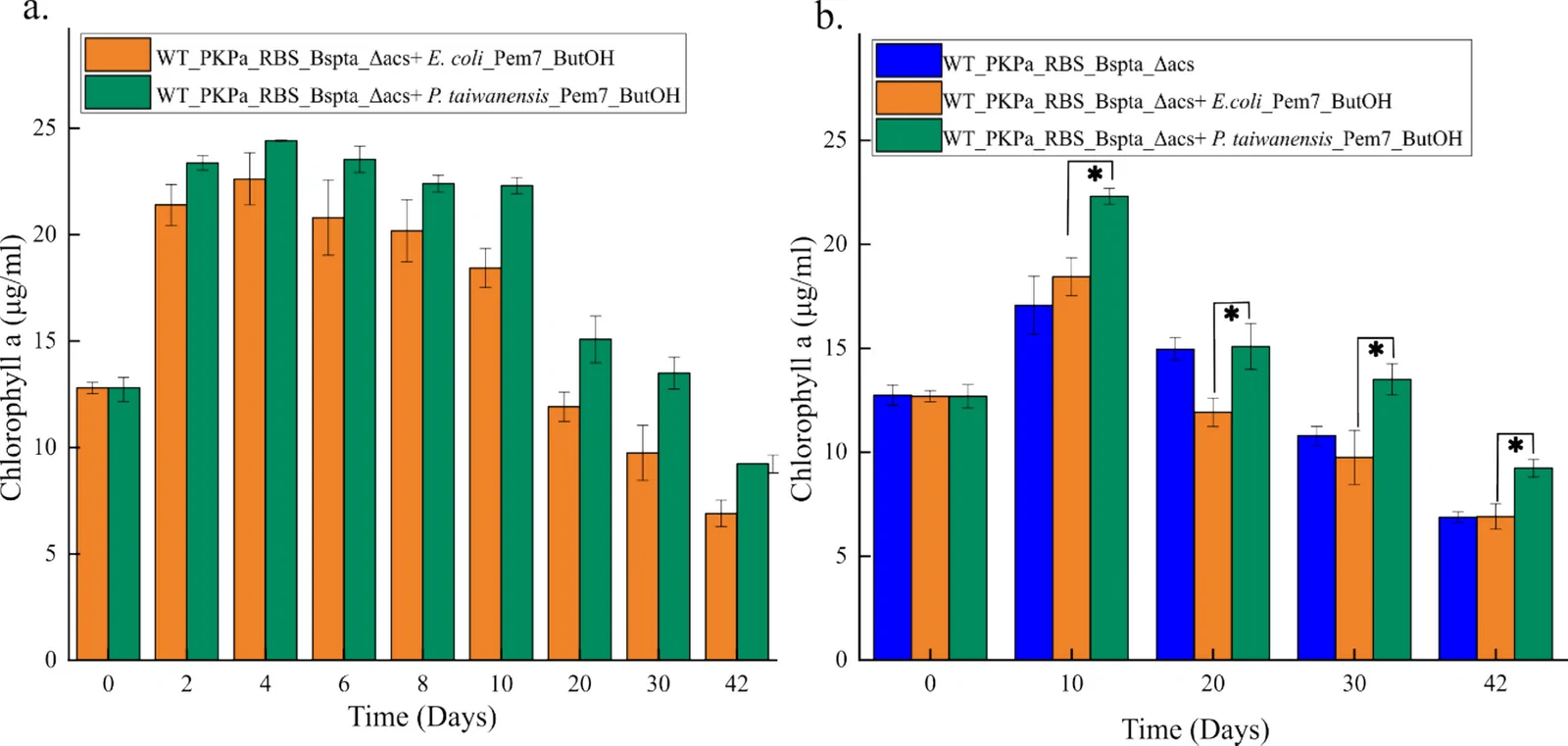
Growth of *Synechocystis* PCC 6803 WT_PKPa_RBS_BsPta_Δacs during the synthetic consortia with either *E. coli*_Pem7_ButOH or *P. taiwanensis*_Pem7_ButOH. The growth is shown through chlorophyll concentration through selected dates of the experiment (**a**) while it was also compared to the monoculture (**b**). 10% of the volume (2.5 ml) was sampled every 2 days from day 0 to day 30 while from day 30 to day 42, the culture was sampled every 4 days and replaced with 2.5 mL of CoBG11 containing 500 mM NaHCO_3_ (final concentration 50 mM) and kanamycin, and the pH was adjusted at 7 with the addition of 37% HCl. The consortia were growing under light intensity 65 µmol photons m^−2^s^−1^ and the temperature was 30 °C. The results are represented as mean ± SD of the three biological replicates

When it comes to the growth of the *Synechocystis* strain WT_PKPa_RBS_BsPta_Δacs in the co-cultures, the chlorophyll a concentration increased between days 2 to 6 followed by a stable period between days 6 and 10 and then started dropping after day 20 (Fig. 8). Between the two synthetic consortia, the chlorophyll a concentration of the WT_PKPa_RBS_BsPta_Δacs strain co-cultivated with *P. taiwanensis*_Pem7_ButOH was consistently higher throughout the experiment, while on days 10 to 42, this difference is also statistically significant. The chlorophyll concentration of days 0, 10, 20, 30, and 42 were also compared to the chlorophyll a concentration on the respective days in the *Synechocystis* monoculture. There, the trend is similar. On days 10, 30, and 42, the difference in the chlorophyll a concentration of the co-cultivation with *P. taiwanensis*_Pem7_ButOH is statistically significantly higher compared to that in the *Synechocystis* cultivated with *E. coli*_Pem7_ButOH. The carotenoid concentrations were similar between the three triplicates (Figure S.3).

**Fig. 9:**
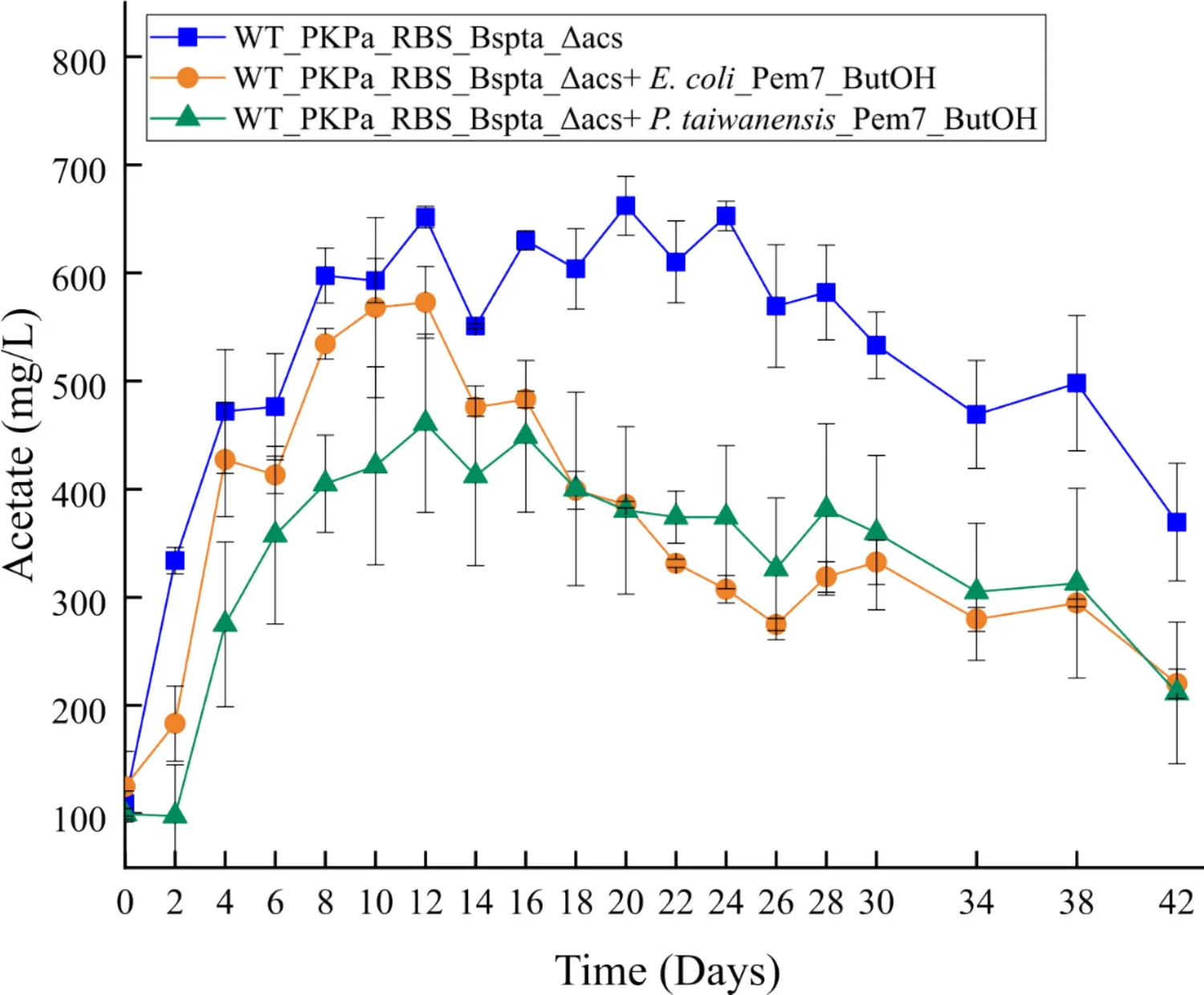
Acetate titers during the synthetic consortia and the monoculture of *Synechocystis* PCC 6803 WT_PKPa_RBS_BsPta_Δacs. The light intensity was 65 μmol photons m^−2^s^−1^ and the temperature was 30 °C. 10% of the volume (2.5 mL) was sampled every 2 days from day 0 to day 30 while from day 30 to day 42, the culture was sampled every 4 days and replaced with 2.5 mL of CoBG11 containing 500 mM NaHCO_3_ (final concentration 50 mM) and kanamycin and the pH was adjusted at 7 with the addition of 37% HCl. On day 0, the heterotrophs were added at the respective triplicate of cyanobacterium and the co-cultivation started. The 2.5 mL was used for OD, acetate measurements, 1-butanol measurements in the consortia, and on specific dates for chlorophyll measurements. The results are represented as mean ± SD of the three biological replicates

**Fig. 10:**
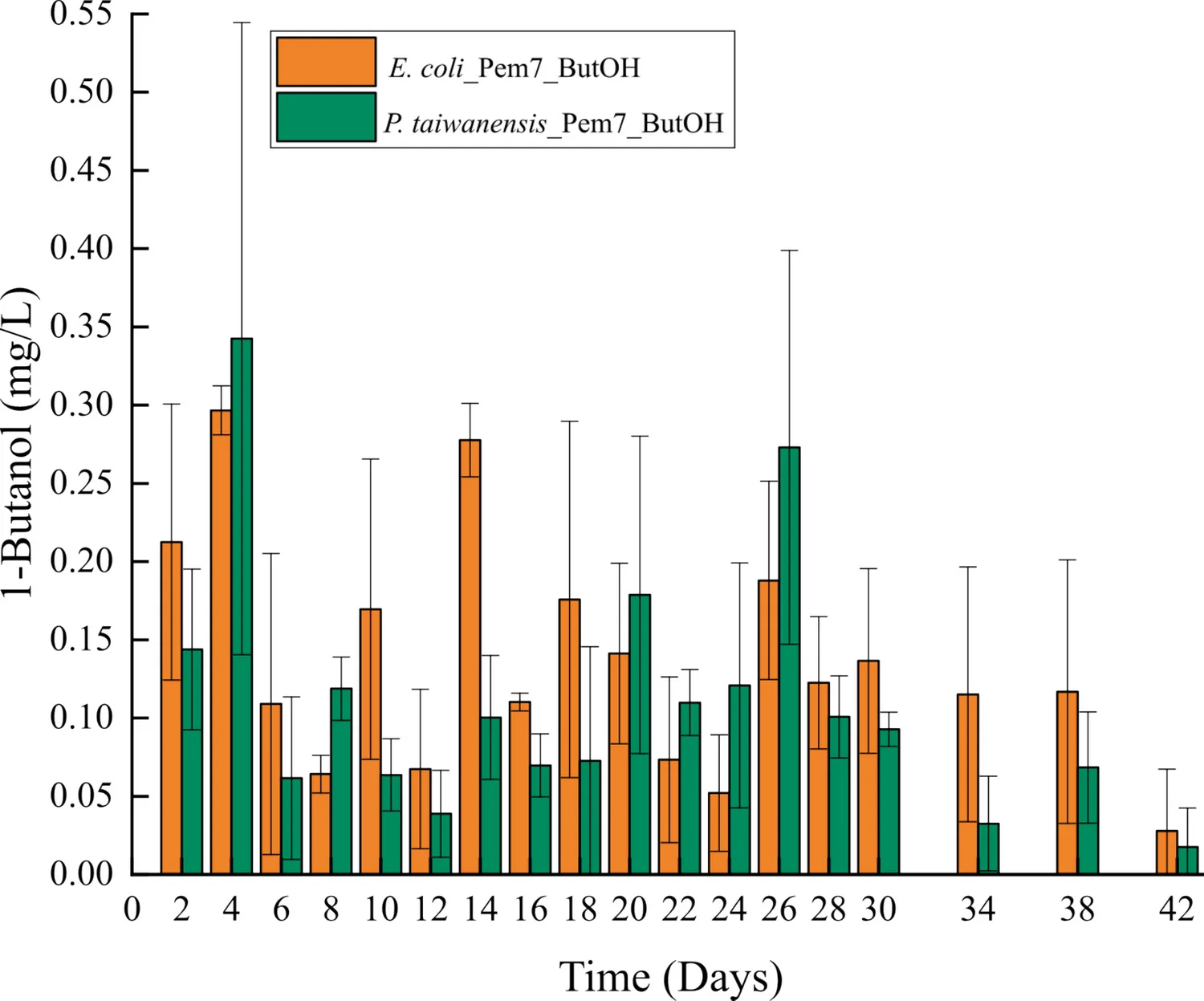
1-Butanol production through the synthetic consortia consisting of *E. coli*_Pem7_ButOH or *P. taiwanensis*_Pem7_ButOH and *Synechocystis* PCC 6803 WT_PKPa_RBS_BsPta_Δacs. The light intensity was 65 μmol photons m^−2^s^−1^ and the temperature was 30 °C. 10% of the volume (2.5 mL) was sampled every 2 days from day 0 to day 30 while from day 30 to day 42, the culture was sampled every 4 days and replaced with 2.5 mL of CoBG11 containing 500 mM NaHCO_3_ (final concentration 50 mM) and kanamycin. On day 0, the heterotrophs were added at the respective triplicate of cyanobacterium and the co-cultivation started. The 2.5 mL was used for OD, acetate measurements, 1-butanol measurements in the consortia, and on specific dates for chlorophyll measurements. The results are represented as mean ± SD of the three biological replicates

The acetate concentration was also measured throughout the experiment (Fig. 9). It is clear that the acetate levels in the *Synechocystis* monoculture remained higher throughout the 42 days of the experiment. The acetate levels showed increased levels until day 8 and after that an overall stability until day 30 followed by a gradual drop until the last day of the experiment. When it comes to the synthetic consortia, the consortium based on *E. coli*_Pem7_ButOH and WT_PKPa_RBS_BsPta_Δacs showed higher acetate than the one based on *P. taiwanensis*_Pem7_ButOH until day 14, and then the values are similar and eventually, after day 22 has a lower acetate concentration. The decrease in acetate is in general sharper in the case of *E. coli*_Pem7_ButOH compared to when *P. taiwanensis*_Pem7_ButOH was the bacterium in the consortium. Finally, the production of 1-butanol was quantified in the synthetic consortia (Fig. 10). It was possible to measure 1-butanol throughout the experiment. However, the production was not stable in the systems but it clearly reached a plateau. The first 4 days of the experiment production of 1-butanol comparable to the one measured in the previous growth experiments were observed. Additionally, on specific days, for example on days 10 and 14, a relatively high production was noted in the consortium containing the *E. coli*_Pem7_ButOH while on days 20 and 26, *P. taiwanensis*_Pem7_ButOH showed high production.

## Discussion

Synthetic consortia have recently gained attention from the scientific community due to their potential advantages. One promising type of consortium is between photosynthetic and heterotrophic microorganisms because the photosynthetic one can take up CO_2_ from the atmosphere and, with energy deriving from sunlight, transform to carbon compounds like sucrose. These compounds can then be used by a heterotroph for the synthesis of a valuable chemical (Hays et al. 2017). In this study, we investigated the potential use of acetate as a carbon source for *E. coli* BL21 and *P. taiwanensis* VLB120. We investigated the ability of these bacteria to grow on acetate as a sole carbon source and also in a consortium with a high-acetate-producing *Synechocystis* strain. The strain is expressing a heterologous phosphoketolase (PKPa) and a heterologous phosphotransacetylase (BsPta) under the same promoter, organized in the same locus of the genome (*acs*) (WT_PKPa_RBS_BsPta_Δacs). The strain showed stable acetate production over long-term cultivation (Roussou and Lindblad 2026).

Acetate can be utilized by both *E. coli* and *P. taiwanensis*, mainly through acetyl-CoA synthetase (Acs). This enzyme catalyzes the transformation of acetate to acetyl-CoA with the parallel consumption of 2 ATP (ATP → AMP). Another pathway is through acetate kinase (AckA) and phosphotransacetylase (Pta). AckA catalyzes the reaction acetate to acetyl-P and then Pta drives the formation of acetyl-CoA. So, this is a two-step reversible reaction, with the consumption of 1 ATP. The acetyl-CoA can then be utilized by the metabolism through the citric acid (TCA) cycle (Ricci et al. 2025).

Beyond the growth on acetate as a carbon source, we also explored the ability of the bacteria to synthesize 1-butanol. For this reason, we selected a relatively simple pathway, deriving from the fatty acid biosynthesis (Pásztor et al. 2015). Another advantage of this pathway is its relatively higher oxygen tolerance, which is an important characteristic for the development of a consortium with oxygen-evolving *Synechocystis* cells. The minimum medium chosen for the growth experiments was CoBG11, a medium that has been used for developing synthetic consortia between cyanobacteria and heterotrophs (Hays et al. 2017). The strain *E. coli*_Pthl_ButOH was grown in CoBG11 with the addition of different concentrations of SoAc. In general, the pattern observed is that the cells grew better when the acetate was at low concentration indicating toxicity caused by the high acetate levels (Gu et al. 2024). 1-Butanol production was observed during all conditions. Our results indicated that feeding 2 or 4 g/L SoAc can benefit 1-butanol production, especially when expressed per OD. So, the cells can grow on acetate as a sole carbon source using an optimized BG11 medium, CoBG11, while maintaining the capability of 1-butanol formation.

In the next step, we decided to study the consumption of a given amount of acetate on day 0. These experiments were also performed in the strain *P. taiwanensis* VLB120. In addition to the ability of the strain to grow on acetate as a sole carbon source, the strain has also shown promising results when co-cultivating with *Synechocystis* (Bozan et al. 2022). *P. taiwanensis*_Pem7_ButOH grew better with 2 g/L SoAc as a carbon source, compared to *E. coli*_Pem7_ButOH. In general, *Pseudomonas* sp. show higher tolerance and acetate utilization compared to *E. coli* (Mutyala et al. 2023). When the cultures were grown with 1 g/L SoAc, growth was visible for both strains, even though *E. coli*_Pem7_ButOH grew better on acetate as the carbon source, probably indicating a faster adaptation in this experimental setup.

Then, adaptive evolution was performed to increase the strain’s capability to utilize acetate. For this reason, the acetate provided was 1 g/L. The adapted strains were then grown provided 1 g/L and 0.5 g/L SoAc. Important to note here is that even though the calculations were made for these values, the measured acetate concentrations on day 0 were actually lower, proving that both of the strains can show growth on very little amount of initial acetate, a promising result for the development of co-cultivation systems. The strains got adapted to grow better on low concentration of acetate; however, the 1-butanol production did not exceed the levels prior to adaptation. One possible explanation for this is that the acetate assimilation for the strain is requiring ATP while not increasing the availability of NAD(P)H, the reducing molecule required for 1-butanol biosynthesis. Another possible explanation is related to the nature of the production pathway, which diverts carbon from the fatty acid biosynthesis (Ricci et al. 2025; Pásztor et al. 2015). Under carbon-limited conditions, fatty acid synthesis may be downregulated, limiting carbon flux through the pathway and restricting 1-butanol production. When it comes to *P. taiwanensis*_Pem7_ButOH 1-butanol production level, the pattern of similar levels between the growth conditions is clear throughout the experiments. The 1-butanol formation seems to be stable across the different media tested for the strain. These results can indicate that the strain grows and efficiently produces 1-butanol under growth on minimum medium with acetate as a sole carbon source, while the low levels could indicate that this pathway is not optimal for the strain. The 1-butanol values per volume and per OD may also show that the strain capacity to produce 1-butanol is not connected to the growth but on the carbon availability through the fatty acid pathway. In the end of this round of experiments, strains of *E. coli* and *P. taiwanensis* with enhanced growth on acetate as a sole carbon source and ability of 1-butanol synthesis were developed.

To develop a synthetic consortium consisting of autotrophic and heterotrophic microorganisms based on acetate as a carbon source, a previously developed acetate-producing *Synechocystis* strain was used as the provider (farmer) strain: WT_PKPa_RBS_BsPta_Δacs. The reason for adjustment of the pH range (to 7) was to ensure that all three different organisms will be cultivated within a living pH range considering the fact that the addition of bicarbonate will eventually shift the pH towards more alkali values. The acetate production in the axenic *Synechocystis* culture did not reach previously reported values (Roussou and Lindblad 2026). One possible explanation may be that the adjustment of pH at 7 was stressful for the cells. Furthermore, the selected medium (CoBG11) contains a relatively high salt concentration (150 mM NaCl), chosen since it has been used to induce sucrose production in *Synechococcus* (Hays et al. 2017). However, it is possible that the elevated salinity may not have been optimal for acetate production in *Synechocystis* resulting in moderate accumulation (Takashima et al. 2020). Another interesting observation in axenic culture was the observed fluctuation in acetate production, most likely caused by the sampling procedure which includes the removal of 10% of culture volume and the subsequent dilution of the culture by the addition of fresh medium (10%). After day 10, the cells are entering stationary phase with a plateau in the production. On the last days of the experiment, roughly after day 24, the production in principle stopped. One theory is that the cells do not produce more acetate so the levels are dropping because of the sampling process.

The initial ratio between the two organisms was 5:1 (*Synechocystis* to heterotrophic). The reasoning was to achieve an optimal equilibrium between the heterotrophic bacteria and the acetate availability. For *E. coli*_Pem7_ButOH, a log phase in the beginning of the experiment is noticeable as the bacterium needed to adjust to both the low acetate level and the co-culture with another microorganism. Compared to the production of acetate in the axenic culture, less amount of acetate was measured in the co-culture with *E. coli* indicating acetate consumption. The growth pattern for *P. taiwanensis*_Pem7_ButOH indicated that this strain is likely better to grow in a consortium with *Synechocystis* with acetate as the source of carbon since the CFU/mL was high on day 2. The drop in cell numbers that followed may be due to too low levels of acetate to support the system. This theory is supported by the initial drop of acetate. Again, the acetate levels remained lower than in the axenic *Synechocystis* culture, indicating its consumption in this co-culture as well. However, since cyanobacteria also secrete carbohydrates and have extracellular polysaccharides, we cannot conclude that the heterotrophic bacteria used acetate as the only source of carbon. Chlorophyll measurements between the two consortia and comparison to the axenic *Synechocystis* culture revealed a similar growth pattern, proving that the *Synechocystis* strain grew within the consortia as well. Interestingly, the growth of the cyanobacteria cells in the consortia with *P. taiwanensis* VBL120 was higher, especially after day 10, compared to the axenic *Synechocystis* culture. This is in agreement with the observed beneficial effect of *Pseudomonas* sp. on the growth of cyanobacterial when grown as co-culture (Bozan et al. 2022; Kratzl et al. 2024). Several strategies can be used to optimize the system, one of them is to explore different initial ratios between the two organisms. This could be feasible for example with the employment of different bioinformatic models (Klitgord and Segrè 2010). Another option can be the optimization or replacement of the 1-butanol production pathway. It would also be interesting to investigate potential evaporation of 1-butanol, even though it is a closed system, as well as the possibility of 1-butanol uptake by the heterotrophs due to low availability of carbon. This study clearly demonstrates the ability of engineered *E. coli* BL21 and *P. taiwanensis* VBL120 to individually grow in consortia on photosynthetically produced acetate and generate an industrially valuable chemical such as 1-butanol.

## Supplementary Information

Below is the link to the electronic supplementary material.

ESM 1 (PDF 281 KB)

## Data Availability

No datasets were generated or analysed during the current study.

## References

[CR1] Abdalla SB, Moghazy RM, Hamed AA, Abdel-Monem MO, El-Khateeb MA, Hassan MG (2024) Strain selection and adaptation of a fungal-yeast-microalgae consortium for sustainable bioethanol production and wastewater treatment from livestock wastewater. Microb Cell Fact 23(1):288. 10.1186/s12934-024-02537-4 39438859 PMC11495080

[CR2] Abinandan S, Subashchandrabose SR, Venkateswarlu K, Megharaj M (2018) Nutrient removal and biomass production: advances in microalgal biotechnology for wastewater treatment. Crit Rev Biotechnol 38(8):1244–1260. 10.1080/07388551.2018.1472066 29768936

[CR3] Arnold S, Henkel M, Wanger J, Wittgens A, Rosenau F, Hausmann R (2019) Heterologous rhamnolipid biosynthesis by *Pseudomonas putida* KT2440 on bio-oil derived small organic acids and fractions. AMB Express 9(1):80. 10.1186/s13568-019-0804-7 31152276 PMC6544668

[CR4] Bolay P, Toepel J, Bühler B (2025) Biotechnological applications of cyanobacteria: *Synechocystis* and *Synechococcus* strains. In: Unconventional Organisms in Biotechnology (pp. 155–191). 10.1007/10_2025_282 40128389

[CR5] Bozan M, Schmid A, Bühler K (2022) Evaluation of self-sustaining cyanobacterial biofilms for technical applications. Biofilm 4:100073. 10.1016/j.bioflm.2022.100073 35434604 PMC9006728

[CR6] Collas F, Kuit W, Clément B, Marchal R, López-Contreras AM, Monot F (2012) Simultaneous production of isopropanol, butanol, ethanol and 2,3-butanediol by *Clostridium acetobutylicum* ATCC 824 engineered strains. AMB Express 2(1):45. 10.1186/2191-0855-2-45 22909015 PMC3583297

[CR7] Du W, Jongbloets JA, van Boxtel C, Pineda Hernández H, Lips D, Oliver BG, Hellingwerf KJ, Branco dos Santos F (2018) Alignment of microbial fitness with engineered product formation: obligatory coupling between acetate production and photoautotrophic growth. Biotechnol Biofuels 11(1):38. 10.1186/s13068-018-1037-8 29456625 PMC5809919

[CR8] Eiteman MA, Lee SA, Altman E (2008) A co-fermentation strategy to consume sugar mixtures effectively. J Biol Eng 2(1):3. 10.1186/1754-1611-2-3 18304345 PMC2266900

[CR9] Gao H, Manishimwe C, Yang L, Wang H, Jiang Y, Jiang W, Jiang M (2022) Applications of synthetic light-driven microbial consortia for biochemical production. Bioresource Technol 351:126954. 10.1016/j.biortech.2022.126954 35288267

[CR10] Großkopf T, Soyer OS (2014) Synthetic microbial communities. Curr Opin Microbiol 18:72–77. 10.1016/j.mib.2014.02.002 24632350 PMC4005913

[CR11] Gu P, Li F, Huang Z, Gao J (2024) Application of acetate as a substrate for the production of value-added chemicals in *Escherichia coli*. Microorganisms 12(2):309. 10.3390/microorganisms12020309 38399713 PMC10891810

[CR12] Hays SG, Yan LLW, Silver PA, Ducat DC (2017) Synthetic photosynthetic consortia define interactions leading to robustness and photoproduction. J Biol Eng 11:4. 10.1186/s13036-017-0048-5 28127397 PMC5259876

[CR13] Huang H-H, Camsund D, Lindblad P, Heidorn T (2010) Design and characterization of molecular tools for a synthetic biology approach towards developing cyanobacterial biotechnology. Nucleic Acids Res 38:2577–2593. 10.1093/nar/gkq164 20236988 PMC2860132

[CR14] Jia X, Liu C, Song H, Ding M, Du J, Ma Q, Yuan Y (2016) Design, analysis and application of synthetic microbial consortia. Synthetic Systems Biotechn 1(2):109–117. 10.1016/j.synbio.2016.02.001 PMC5640696 29062933

[CR15] Jin C, Yao M, Liu H, Lee C-FF, Ji J (2011) Progress in the production and application of n-butanol as a biofuel. Renew Sustain Energy Rev 15(8):4080–4106. 10.1016/j.rser.2011.06.001

[CR16] Keller L, Surette MG (2006) Communication in bacteria: an ecological and evolutionary perspective. Nat Rev Microbiol 4(4):249–258. 10.1038/nrmicro1383 16501584

[CR17] Kiefer D, Merkel M, Lilge L, Henkel M, Hausmann R (2021) From acetate to bio-based products: underexploited potential for industrial biotechnology. Trends Biotechnol 39(4):397–411. 10.1016/j.tibtech.2020.09.004 33036784

[CR18] Klitgord N, Segrè D (2010) Environments that induce synthetic microbial ecosystems. PLoS Comput Biol 6(11):e1001002. 10.1371/journal.pcbi.1001002 21124952 PMC2987903

[CR19] Kratzl F, Urban M, Pandhal J, Shi M, Meng C, Kleigrewe K, Pflüger-Grau K (2024) *Pseudomonas putida* as saviour for troubled *Synechococcus elongatus* in a synthetic co-culture: interaction studies based on a multi-omics approach. Commun Biol 7(1):452. 10.1038/s42003-024-06098-5 38609451 PMC11014904

[CR20] Liang F, Lindberg P, Lindblad P (2018) Engineering photoautotrophic carbon fixation for enhanced growth and productivity. Sustain Energy Fuels 2(12):2583–2600. 10.1039/C8SE00281A

[CR21] Liu X, Miao R, Lindberg P, Lindblad P (2019) Modular engineering for efficient photosynthetic biosynthesis of 1-butanol from CO₂ in cyanobacteria. Energy Environ Sci 12(9):2765–2777. 10.1039/C9EE01214A

[CR22] Marchand N, Collins CH (2013) Peptide-based communication system enables *Escherichia coli* to *Bacillus megaterium* interspecies signaling. Biotechnol Bioeng 110(11):3003–3012. 10.1002/bit.24975 23775238

[CR23] Martin BD, Schwab E (2013) Current usage of symbiosis and associated terminology. Int J Biol 5(1):32. 10.5539/ijb.v5n1p32

[CR24] Melis A, Hidalgo Martinez DA, Betterle N (2024) Perspectives of cyanobacterial cell factories. Photosynth Res 162(2):459–471. 10.1007/s11120-023-01056-4 37966575 PMC11615099

[CR25] Mutalik VK, Guimaraes JC, Cambray G, Lam C, Christoffersen MJ, Mai QA, Tran AB, Paull M, Keasling JD, Arkin AP, Endy D (2013) Precise and reliable gene expression via standard transcription and translation initiation elements. Nat Methods 10(4):354–360. 10.1038/nmeth.2404 23474465

[CR26] Mutyala S, Li S, Khandelwal H, Kong DS, Kim JR (2023) Citrate synthase overexpression of *Pseudomonas putida* increases succinate production from acetate in microaerobic cultivation. ACS Omega 8(29):26231–26242. 10.1021/acsomega.3c02520 37521642 PMC10373214

[CR27] Neves D, Meinen D, Alter TB, Blank L, Ebert BE (2024) Expanding *Pseudomonas taiwanensis* VLB120’s acyl-CoA portfolio: propionate production in mineral salt medium. Microb Biotechnol 17(1):e14309. 10.1111/1751-7915.14309 37537795 PMC10832534

[CR28] Noh MH, Lim HG, Woo SH, Song J, Jung GY (2018) Production of itaconic acid from acetate by engineering acid-tolerant *Escherichia coli* W. Biotechnol Bioeng 115(3):729–738. 10.1002/bit.26508 29197183

[CR29] Pásztor A, Kallio P, Malatinszky D, Akhtar MK, Jones PR (2015) A synthetic O₂-tolerant butanol pathway exploiting native fatty acid biosynthesis in *Escherichia coli*. Biotechn Bioeng 112(1):120–128. 10.1002/bit.25324 24981220

[CR30] Pathania R, Srivastava A (2025) Advances in organic acid production using cyanobacteria: strategies and applications. Blue Biotechn 2(1):1–19. 10.1186/s44315-025-00045-7

[CR31] Phue JN, Lee SJ, Kaufman JB, Negrete A, Shiloach J (2010) Acetate accumulation through alternative metabolic pathways in *Escherichia coli* B (BL21). Biotechn Lett 32(12):1897–1903. 10.1007/s10529-010-0369-7 PMC9554928 20703804

[CR32] Qian X, Chen L, Sui Y, Chen C, Zhang W, Zhou J, Ochsenreither K (2020) Biotechnological potential and applications of microbial consortia. Biotechnol Adv 40:107500. 10.1016/j.biotechadv.2019.107500 31862233

[CR33] Ricci L, Cen X, Zu Y, Antonicelli G, Chen Z, Fino D, Pirri FC, Stephanopoulos G, Woolston BM, Re A (2025) Metabolic engineering of *E. coli* for enhanced diols production from acetate. ACS Synth Biol 14(4):1204–1219. 10.1021/acssynbio.4c00839 40103233 PMC12012870

[CR34] Ritchie RJ (2006) Consistent sets of spectrophotometric chlorophyll equations for acetone methanol and ethanol solvents. Photosynth Res 89(1):27–41. 10.1007/s11120-006-9065-9 16763878

[CR35] Roussou S, Lindblad P (2026) Increased acetate production in *Synechocystis* sp. PCC 6803 strain engineered with an operon of phosphoketolase and phosphotransacetylase and further overexpression of acetate kinase. Microb Cell Fact 25:61. 10.1186/s12934-026-02964-5 41709309 PMC12930666

[CR36] Roussou S, Pan M, Krömer JO, Lindblad P (2025) Exploring and increase of acetate biosynthesis in *Synechocystis* PCC 6803 through insertion of a heterologous phosphoketolase and analyses of the native key pathway. Metab Eng 88:250–260. 10.1016/j.ymben.2025.01.008 39863056

[CR37] Shong J, Diaz MR, Collins CH (2012) Towards synthetic microbial consortia for bioprocessing. Curr Opin Biotech 23(5):798–802. 10.1016/j.copbio.2012.02.001 22387100

[CR38] Slusarczyk AL, Lin A, Weiss R (2012) Foundations for the design and implementation of synthetic genetic circuits. Nat Rev Genet 13(6):406–420. 10.1038/nrg3227 22596318

[CR39] Stanier RY, Kunisawa R, Mandel M, Cohen-Bazire G (1971) Purification and properties of unicellular blue-green algae (order Chroococcales). Bacteriol Rev 35(2):171–205. 10.1128/br.35.2.171-205.1971 PMC378380 4998365

[CR40] Takashima K, Nagao S, Kizawa A, Suzuki T, Dohmae N, Hihara Y (2020) The role of transcriptional repressor activity of LexA in salt-stress responses of the cyanobacterium *Synechocystis* sp. PCC 6803. Sci Rep 10(1):17393. 10.1038/s41598-020-74534-7 33060671 PMC7567884

[CR41] Tóth GS, Siitonen V, Nikkanen L, Sovic L, Kallio P, Kourist R, Allahverdiyeva Y (2022) Photosynthetically produced sucrose by immobilized *Synechocystis sp.* PCC 6803 drives biotransformation in *Escherichia coli*. Biotechn Biofuels Bioproducts 15(1):146. 10.1186/s13068-022-02248-1 PMC9795604 36575466

[CR42] Wang X, Jin M, Balan V, Jones AD, Li X, Li BZ, Yuan YJ (2014) Comparative metabolic profiling revealed limitations in xylose-fermenting yeast during co-fermentation of glucose and xylose in the presence of inhibitors. Biotechnol Bioeng 111(1):152–164. 10.1002/bit.24992 24404570

[CR43] Wellburn AR (1994) The spectral determination of chlorophylls a and b, as well as total carotenoids, using various solvents. J Plant Physiol 144(3):307–313. 10.1016/S0176-1617(11)81192-2

[CR44] Zhang L, Chen L, Diao J, Song X, Shi M, Zhang W (2020) Construction and analysis of an artificial consortium based on *Synechococcus elongatus* UTEX 2973 to produce 3-hydroxypropionic acid from CO₂. Biotechnol Biofuels 13(1):1–14. 10.1186/s13068-020-01720-0 32391082 PMC7201998

[CR45] Zhu J, Liu W, Guo L, Tan X, Sun W, Zhang H, Tian W, Jiang T, Meng W, Liu Y, Kang Z, Gao C, Lü C, Xu P, Ma C (2024) Acetate production from corn stover hydrolysate using recombinant *Escherichia coli* BL21 (DE3) with an EP-bifido pathway. Microb Cell Fact 23:300. 10.1186/s12934-024-02575-y 39523316 PMC11552437

[CR46] Zobel S, Benedetti I, Eisenbach L, de Lorenzo V, Wierckx N, Blank LM (2015) Tn7-based device for calibrated heterologous gene expression in *Pseudomonas putida*. ACS Synth Biol 4(12):1341–1351. 10.1021/acssynbio.5b00058 26133359

