# Supplementary material for: Synthetic consortia towards photosynthetically derived acetate for heterotrophic production of 1-butanol
Source: Appl Microbiol Biotechnol. 2026 Sep 11;110:268. doi: 10.1007/s00253-026-14029-z (PMC13569817; doi:10.1007/s00253-026-14029-z)
Supplement: Supplementary file 1 [file 253_2026_14029_MOESM1_ESM.pdf]

SUPPLEMENTARY

Synthetic consortia towards photosynthetically derived acetate for heterotrophic production of 1-butanol

Stamatina Roussou and Peter Lindblad*

stamatina.roussou@kemi.uu.se

peter.lindblad@kemi.uu.se

Microbial Chemistry, Department of Chemistry-Ångström Laboratory, Uppsala University, Box 523, SE-75120

Uppsala, Sweden

* Corresponding author

Appendix A

Tables S.1-S.2

Figures S.1-S.3

Table S.1 Primers (a) and gBlock (b) used in this study

a. Primers

Name	DNA sequence	Reference
Pthl_XbaI_F	GCCGCTTCTAGAGGCAGCCAATAAGAAGGAGAAG	This study
Pthl_OEBF_R	CTCCCAATTTTTTTGTCATCGCTCATCTTGTCATCGTCATCCTTGTA	This study
BF_Pthl_F	ATGAGCGATGACAAAAAAATT	This study
BF_colony_R	GGCCATTTCAAAGCGCTGAA	This study
ppt_end_F	ATCCGAATTCAGGAGGTAAAAAAATGAGCC	This study
ppt_end_R	ATGGCTCATTTTTTTACCTCCTGAATTCGG	This study
SpeI_car_R	CCGCTACTAGTATTACAGCAGGCCCAGCAG	This study
VR_SR_R	AACCGAAGTAATCTTTT	This study
RSF1010_seq	CCCTGCTGGGTTTGCAGG	This study
SRV_seq_R	GCTGGACAGCGAAACCGC	This study
VR_SR_F	AAAATAGGCGTATCACGAG	This study
car_colony_F	TGAATTTGTGGATTGGCTGA	This study

b. gBlock for Pem7

GCAGCCAATAAGAAGGAGAAGGCGATTGTTGACAATTAATCATCGGCATAGTATATCGG
CATAGTATAATACGACAAGGTGAGGAACTAAACCCCTAGGGCCCAAGTTCACTTAAAAA
GGAGATCAACAATGAAAGCAATTTTCGTACTGAAACATCTTAATCATGCTAAGGAGGTTT
TCTAATGGACTACAAGGATGACGATGACA

With Red the Pem7 promoter is highlighted

With Green the BCD2 region is highlighted

With Blue the Flag Taq is highlighted

Table S.2 Plasmids and strains used in this study

Plasmid	Promoters and genes encoding for the enzymes	Strains	References
pPMQAK1	*Empty vector*	*E. coli*_pPMQAK1	Huang et al. 2010
pET-TPC4	*PT7*, aat_{BF}*-ppt_{BS}*[1]-car_{MM}*[2]	*E. coli*_pET_TPC4	Pásztor et al. 2015
Pthl_ButOH	*Pthl*, aat_{BF}, ppt_{BS}, car_{MM}	*E. coli*_Pthl_ButOH<br>*P. taiwanensis*_Pthl_ButOH	This study
Pem7_ButOH	*Pem7*, aat_{BF}, ppt_{BS}, car_{MM}	*E. coli*_Pem7_ButOH<br>*P. taiwanensis*_Pem7_ButOH	This study

*aat_{BF}: gene encoding for acyl-ACP thioesterase from *Bacteroides fragilis*

*[1]ppt_{BS}: gene encoding for phosphopantetheinyl trasferase maturation factor from *Bacillus subtilis*

*[2]car_{MM}: gene encoding for carboxylic acid reductase from *Mycobacterium marinum*

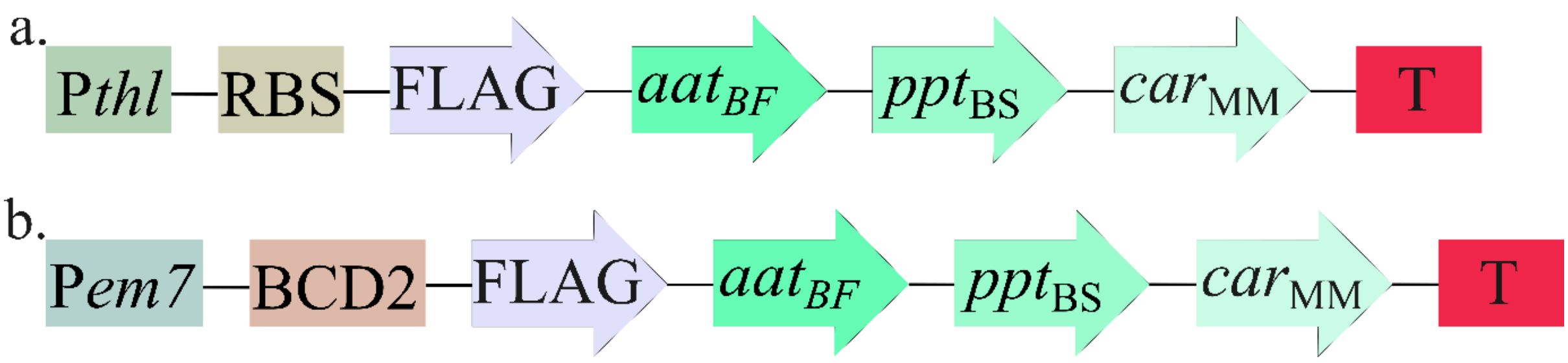

Figure S.1: Schematic representation of the genetic constructs developed in this study. P*thl* (a) and P*em7* (b) represents the two different promoters tested in this study. In (a), an RBS is followed, the Flag taq and the genetic construct for the expression of the 1-butanol pathway. The genes included are *aat*$_{BF}$; acyl-ACP thioesterase from *Bacteroides fragilis*, *ppt*$_{BS}$; phosphopantetheinyl transferase maturation factor from *Bacillus subtilis* and *car*$_{MM}$; carboxylic acid reductase from *Mycobacterium marinum.* The T represents his operon terminator located on the pPMQAK1 plasmid. In (b), the BCD2 region is following the P*em7* promoter instead of the RBS. The rest of the construct remained the same.

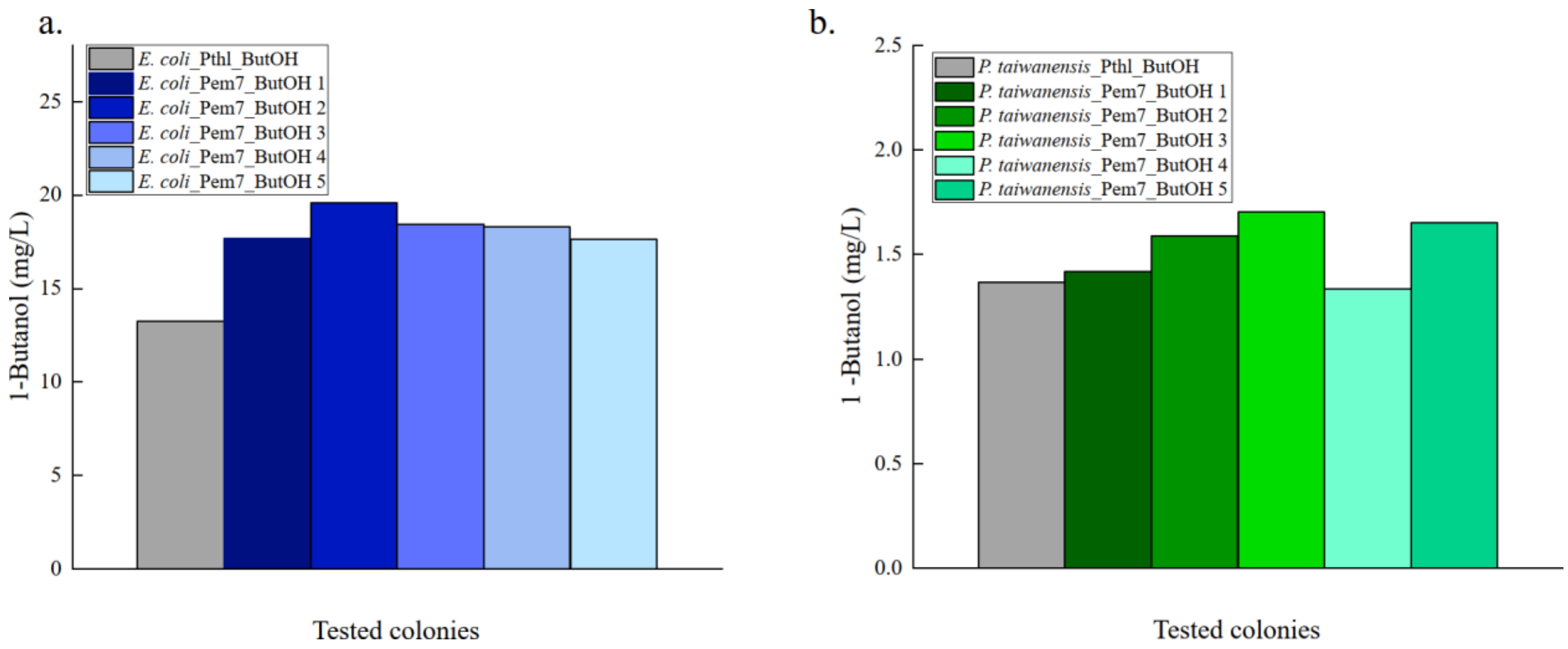

Figure S.2: Screening experiment for individual colonies of *E. coli* BL21 (a) and *P. taiwanensis* VBL120 (b) after transformation with Pem7_ButOH compared to strains containing the plasmid Pthl_ButOH. The colonies were grown in a closed system overnight and then the 1-butanol production was quantified.

a.

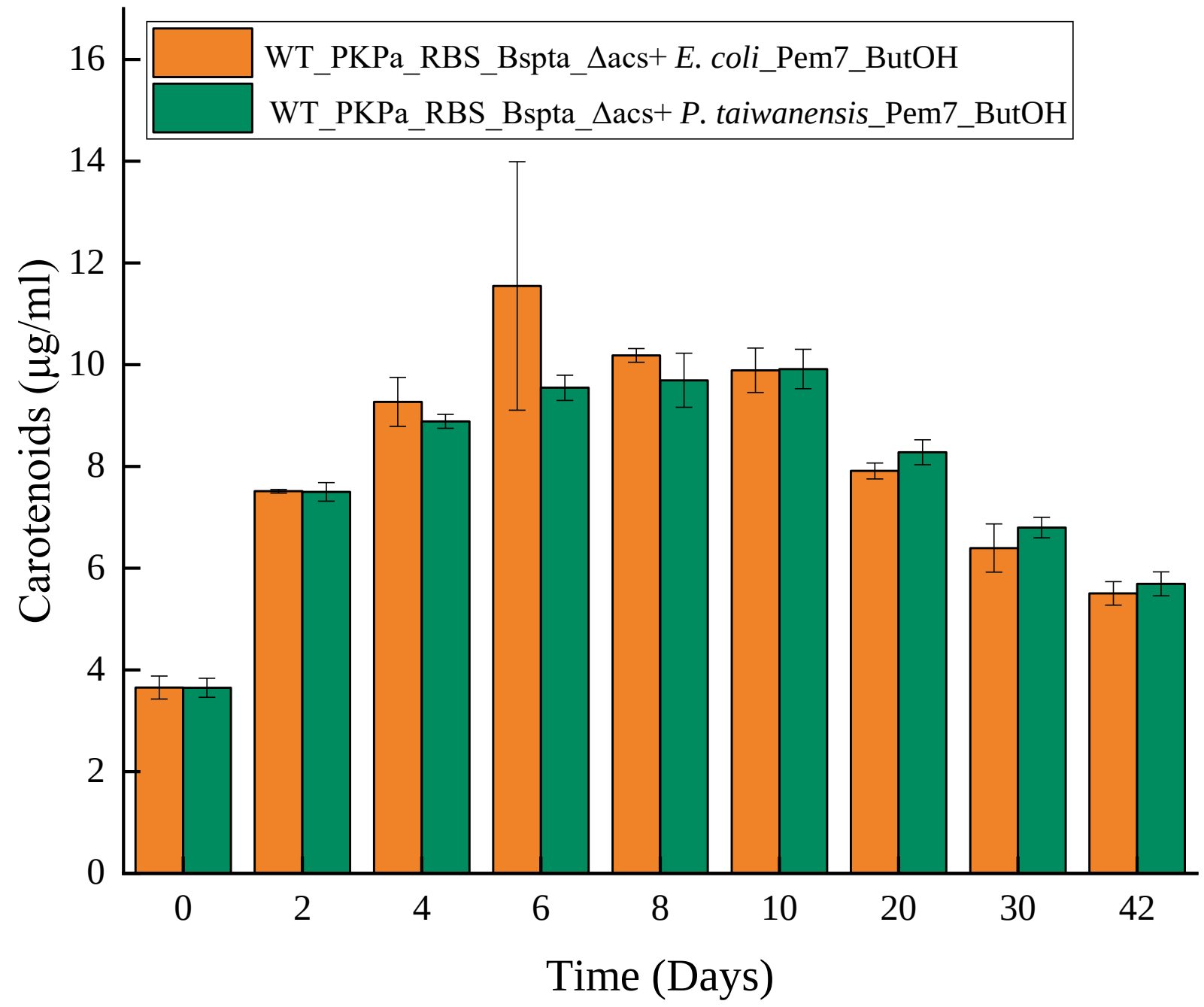

b.

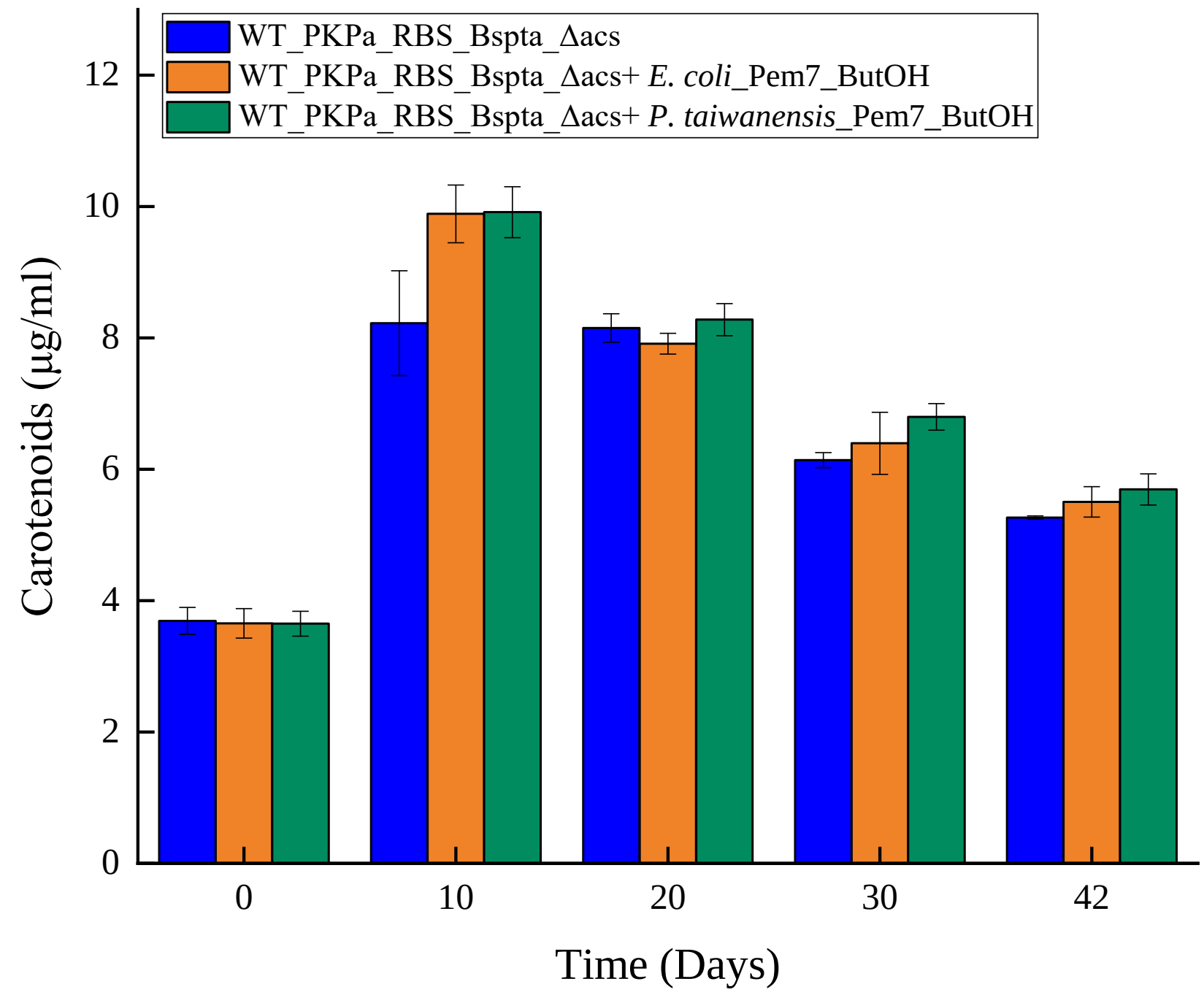

Figure S.3: Carotenoid concentration of *Synechocystis* PCC 6803 WT_PKPa_RBS_BsPta_Δacs (blue) grown in synthetic consortia with either *E. coli*_Pem7_ButOH (orange) or *P. taiwanensis*_Pem7_ButOH (green). The carotenoid concentration is shown through selected dates of the experiment (a) while it was also compared to the monoculture (b). 10% of the volume (2.5 ml) was sampled every 2 days

from day 0 to day 30 while from day 30 to day 42 the culture was sampled every 4 days and replaced with 2.5 ml of CoBG11 containing 500 mM $NaHCO_3$ (final concentration 50 mM) and kanamycin and the pH was adjusted at 7 with the addition of 37% HCl. The consortia were growing under light intensity 65 μmol photons m^{-2} s^{-1} and the temperature was 30ºC. The results are represented as mean ± SD of the three biological replicates.

References

Huang H-H, Camsund D, Lindblad P, Heidorn T (2010) Design and characterization of molecular tools for a synthetic biology approach towards developing cyanobacterial biotechnology. *Nucleic Acids Res 38*:2577–2593. https://doi.org/10.1093/nar/gkq164

Pásztor A, Kallio P, Malatinszky D, Akhtar MK, Jones PR (2015) A synthetic O_2-tolerant butanol pathway exploiting native fatty acid biosynthesis in *Escherichia coli*. *Biotechn Bioeng 112*(1):120–128. https://doi.org/10.1002/bit.25324
